# Molecular detection of per- and polyfluoroalkyl substances in water using time-of-flight secondary ion mass spectrometry

**DOI:** 10.3389/fchem.2023.1253685

**Published:** 2023-10-06

**Authors:** Xiao-Ying Yu, Cuiyun Yang, Jun Gao, John Xiong, Xiao Sui, Lirong Zhong, Yuchen Zhang, Jiyoung Son

**Affiliations:** ^1^ Oak Ridge National Laboratory, Materials Science and Technology Division, Oak Ridge, TN, United States; ^2^ Haley & Aldrich Inc., Costa Mesa, CA, United States; ^3^ College of Geography and Environment, Shandong Normal University, Jinan, China; ^4^ Pacific Northwest National Laboratory, Energy and Environment Directorate, Richland, WA, United States

**Keywords:** PFAS, PFOs, molecular identification, ToF-SIMS, groundwater, wastewater

## Abstract

Detection of per- and polyfluoroalkyl substances (PFASs) is crucial in environmental mitigation and remediation of these persistent pollutants. We demonstrate that time-of-flight secondary ion mass spectrometry (ToF-SIMS) is a viable technique to analyze and identify these substances at parts per trillion (ppt) level in real field samples without complicated sample preparation due to its superior surface sensitivity. Several representative PFAS compounds, such as perfluorooctanesulfonic acid (PFOS), perfluorobutanoic acid (PFBA), perfluoropentanoic acid (PFPeA), perfluoheptanoic acid (PFHpA), and perfluorononanoic acid (PFNA), and real-world groundwater samples collected from monitoring wells installed around at a municipal wastewater treatment plant located in Southern California were analyzed in this work. ToF-SIMS spectral comparison depicts sensitive identification of pseudo-molecular ions, characteristic of reference PFASs. Additionally, principal component analysis (PCA) shows clear discrimination among real samples and reference compounds. Our results show that characteristic molecular ion and fragments peaks can be used to identify PFASs. Furthermore, SIMS two-dimensional (2D) images directly exhibit the distribution of perfluorocarboxylic acid (PFCA) and PFOS in simulated mixtures and real wastewater samples. Such findings indicate that ToF-SIMS is useable to determine PFAS compounds in complex environmental water samples. In conclusion, ToF-SIMS provides simple sample preparation and high sensitivity in mass spectral imaging, offering an alternative solution for environmental forensic analysis of PFASs in wastewater in the future.

## Graphical Abstract

**Figure:**
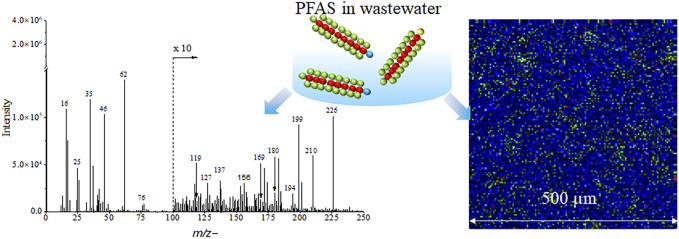

## 1 Introduction

Per- and polyfluoroalkyl substances (PFASs) are a group of manmade synthetic organic fluorinated substances. They have been widely used in industrial and commercial applications for more than 50 years. Representative examples of PFASs applications include surfactants, flame retardants, food packaging, and non-stick coating for cooking utensils (Rahman et al., 2014; Ciccotelli et al., 2016; Monge Brenes et al., 2019; Alves et al., 2020; Clarity et al., 2021). PFASs are ubiquitous in the environment and they have become a global pollution problem, especially perfluorooctanoate (PFOA) and perfluorooctanoate sulfonate (PFOS) due to their persistence, bio-accumulative properties, and toxicities even at low concentrations in the environment (Ciccotelli et al., 2016; Kucharzyk et al., 2017). More than 5,000 individual PFASs have been found in waters, solids, and fish; and many of them are potential precursor compounds of PFOA and PFOS (Chan et al., 2009; Llorca et al., 2009; Xiao, 2017; Dauchy, 2019; Abunada et al., 2020; Chohan et al., 2021; Lassalle et al., 2021). Therefore, studies on PFASs, including PFOA and PFOS, are extremely important to understand the distribution, transformation, and ultimately removal of these persistent organic pollutants from the natural water environment.

PFASs are found in different aqueous matrices including surface water, groundwater, drinking water, lake and costal water, or sea waters (Rayne and Forest, 2009; Crone et al., 2019). Pre-treatment methods, such as filtration and centrifugation, were used for analysis (Nzeribe et al., 2019; Vu and Wu, 2022). At present, the main techniques used to analyze the distribution and compositions of PFASs in the natural environment, food, animal’s blood, or tissue are gas chromatography mass spectrometry (GC-MS) or tandem mass spectrometry (GC-MS/MS), high performance liquid chromatography mass spectrometry or tandem mass spectrometry (HPLC-MS or HPLC-MS/MS), and ultra-high performance liquid chromatography mass spectrometry or tandem mass spectrometry (UPLC-MS or UPLC-MS/MS) (Capriotti et al., 2013; Bach et al., 2016; Mulabagal et al., 2018; Groffen et al., 2021; Qi et al., 2021). These methods are quantitative; however, sample preparation can be challenging. PFAS identification depends heavily on standards and reference chemicals. For example, fluorotelomer alcohols (FTOHs) were often determined by GC-MS, and trace levels of FTOHs are detectable in river water, influent and effluent wastewater samples using silica normal-phase solid phase extraction (SPE) (Portoles et al., 2015; Bach et al., 2016). HPLC and UPLC-MS/MS are currently widely used to determine PFASs (Capriotti et al., 2013; Mulabagal et al., 2018). In addition to these methods, LC-MS/MS is recommended by the United States Environmental Protection Agency (EPA) for PFAS analysis (Benskin et al., 2007; Stramenga et al., 2021). Specifically, Liquid Chromatography Quadrupole Time of Flight tandem Mass Spectrometry (LC-QToF/MS) is used to analyze and identify PFAS in serum samples of firefighters who are exposed to fire extinguishing agents containing PFASs (Rotander et al., 2015). However, these methods need a complex pretreatment procedure to extract or transfer PFASs as derivatives before analysis. Therefore, new analytical approaches that offer easy sample preparation and sensitive detection are attractive to the community of PFAS research and environmental protection and restoration.

Unlike the commonly used MS tools as a bulk analysis approach, time-of-flight secondary ion mass spectrometry (ToF-SIMS) is a powerful, high-resolution surface analysis tool. It provides sensitive spectral mapping of molecular, elemental, and isotopic characteristics of solid samples (Fu et al., 2017; Čižinauskas et al., 2017). Because SIMS measurements have superior surface sensitivity, only a minute amount of sample like microgram or less is needed. Therefore, it does not demand huge amount of mass to perform an analysis, and it often used in trace analysis almost nondestructively. Another attractive feature of SIMS is high mass resolution, often several thousand or ten thousand of relative mass accuracy of detected peaks could be obtained compared to other bulk MS approaches (Gilmore and Seah, 2000; Gilmore et al., 2005). Thus, it offers comprehensive information and sensitive analysis of specimens with spatial distribution in one-dimensional (1D) spectra as well as 2D and 3D mass spectral images (Yu, 2020). Although ToF-SIMS is semi-quantitative, the sample preparation and analysis are simple and fast, yet offering high mass accuracy and high mass resolving power of organic molecules (Touboul et al., 2005; Vickerman and Winograd, 2015; Wei et al., 2017; Zhang et al., 2019b). Full spectral (i.e., elemental, molecular, isotopic) information is available due to the parallel collection nature of ToF-SIMS (Sodhi, 2004; Smentkowski et al., 2007; Fisher et al., 2016). Moreover, ToF-SIMS is known for its applications in forensic analysis, namely, sensitive detection of spectral signatures in minute specimens due to its superior surface sensitivity (Szynkowska et al., 2013; Terlier et al., 2020; Szynkowska-Jóźwik et al., 2021). Also, the contrast in PFASs between surface and deeper soil samples could be more pronounced in long-chain congers than shorter chains (Washington et al., 2010), which presents opportunities for applications of SIMS. Amendment materials such as clay and resin have been developed and used to treat PFAS pollution (Nzeribe et al., 2019; Anderson et al., 2021; Kurwadkar et al., 2022). Once loaded, the fate and transformation of PFAS need investigation (DOD, 2022). These are the primary drivers of knowing the spatial resolution of PFAS in a material. ToF-SIMS potentially provides a new imaging solution to addressing the PFAS challenges.

In this work, we demonstrate that ToF-SIMS can be used for rapid analysis and identification of PFASs using several representative PFAS compounds as examples, and their corresponding pseudo-molecular ion and fragment peaks are observable in this work. Limit of detections (LODs) of representative PFAS molecules, namely, PFPeA and PFOS, were determined. Additionally, 2D imaging offers the possibility to visualize PFASs spatial distribution in a mixture, identifying short-chain and long-chain components in a mixture. Moreover, real-world groundwater samples collected in a wastewater treatment plant (WWTP) were analyzed to demonstrate the sensitive ToF-SIMS detection and identification of PFAS compounds. Our results present the first experimental evidence that ToF-SIMS can be a useful tool for analysis of trace level of PFASs pollutants in laboratory-prepared simulated mixtures as references and in field collected groundwater samples as validation.

## 2 Materials and methods

### 2.1 Chemical agents

Four perfluorocarboxylic acid (PFCA) compounds including perfluorobutanoic acid (PFBA, 95%), perfluoropentanoic acid (PFPeA, 97%), perfluoheptanoic acid (PFHpA, 99%), perfluorononanoic acid (PFNA, 97%), and perfluorooctane sulfonate (PFOS, ∼40% in H_2_O) were acquired from Sigma-Aldrich and they were used as reference materials. Additional descriptions of all chemical reagents and the sample preparation protocol were summarized in Supplementary Table S1.

### 2.2 Field groundwater sample collection

The field samples were collected from a municipal WWTP located in Southern California. The MW-6 and MW-5 groundwater samples were collected from two groundwater monitoring wells installed around the WWTP, using dedicated Groundfos submersible pumps, stored in polypropylene sample bottles with Teflon**®**-free caps, and in compliance with the PFAS sampling guidance document published by the California State Water Resources Control Board (SWRCB). The PFASs of the field samples were determined to be at the ppt level using the EPA recommended Draft Method 1633 (EPA, 2022) by a commercial certified by the Department of Defense laboratory.

### 2.3 Sample preparation

Several reference PFAS chemicals, including PFOS, and groundwater samples were prepared by simply drying the liquid mixtures on clean 1 × 1 cm^2^ silicon (Si) wafer chips after depositing 25 µL of the liquid containing PFAS chemicals on the clean Si chip under ambient conditions (Wei et al., 2017; Fu et al., 2018; Sui et al., 2018). Samples were dried in a laminar flow and protected under Parafilm prior to analysis.

### 2.4 ToF-SIMS

A ToF-SIMS V spectrometer (IONTOF GmbH, Münster, Germany) was used to analyze representative PFAS reference chemicals and PFAS-containing groundwater samples. The SIMS analysis was performed using a 25 keV pulsed bismuth (Bi_3_^+^) primary beam ion under high vacuum of 10^–8^ mbar during measurements. The Bi_3_^+^ primary ion beam scanned over a 500 × 500 μm^2^ area for field water samples and 200 × 200 μm^2^ area for reference chemicals, respectively, with a resolution of 128 by 128 pixels. The pulsed current of Bi_3_^+^ was set at 0.54 pA at a repeating frequency of 10 kHz. Each spectrum was acquired for 100 scans. The primary ion doses in all measurements were lower than the static limit, and the damage artifacts resulting from the Bi_3_^+^ primary ion beam was negligible. Mass resolution was in the range of 3000–7000, varying from sample to sample depending on the sample roughness. At least five positive and five negative ion replicate spectra were collected at various locations randomly for each sample including groundwater samples.

ToF-SIMS spectral analysis and 2D image reconstruction were performed using the IONTOF SurfaceLab 7 software. Mass spectra were calibrated using CH^+^, CH_2_^+^, CH_3_^+^, C_2_H_5_^+^, C_3_H_5_^+^, Si_2_C_5_H_15_O^+^, and Si_3_C_7_H_21_O_2_^+^ in the positive ion mode and CH^−^, C_2_^−^, C_2_H^−^, C_3_H^−^, and SiO_2_^−^ in the negative ion mode, respectively. Results were exported and plotted in Igor 8.0. Interference peaks such as Si were removed before running principal component analysis (PCA). Peaks were selected using spectral overlay. Selected peaks were used in PCA using Matlab (R2020a, Math Works, Inc., United States). SIMS spectral data were treated by normalization to the total ion intensity of selected peaks, square root transformation, and mean-centering prior to performing spectral PCA. More details were available in previous reports (Ding et al., 2016; Zhang et al., 2019a; Wei et al., 2020).

## 3 Results and discussion

### 3.1 LOD determination

It is assumed that the instrument response counts (y) are linearly related to the standard concentration (x) for a limited range of concentration when there is a linear calibration curve (Armbruster and Pry, 2008). This model is used to compute the LOD. The LOD can be expressed as $LOD=3S_{a}/b$ . $S_{a}$ is the standard deviation of the response and b the slope of the calibration curve. The response can be estimated by the standard deviation of either y-residuals, or *y* intercept, of regression lines (Shrivastava and Gupta, 2011; Yu et al., 2020). The LODs were estimated based on the data and linear regressions fits (Supplementary Figures S1, S2), when using SIMS to quantify low concentration (≤1% usually) species (Médard et al., 2002; Coullerez et al., 2003). It is worth noting that six different concentrations of PFOS and PFPeA solutions were analyzed, and a linear relationship was obtained in the low concentration range (see Supplementary Figure S1). This result indicates that the assumption of a linear relationship between signal intensities and concentrations is reasonable. The LODs of PFPeA and PFOS were determined to be 28 and 5.6 ppm, respectively, using 25 μL sample deposition and the standard bunch mode spectral collection conditions.

The LOD can be calculated as $LOD=3S_{a}/b$ . Using this formula, the LOD is determined to be 27.97 mg/L for *m/z* ^−^ 168.994 and 5.59 mg/L for *m/z* ^−^ 268.980. The limit of quantification (which they call QL, the quantitation limit) LOQ can be calculated as $LOQ=10S_{a}/b$ . Using this formula, the LOQ is determined to be 93.23 mg/L for *m/z* ^−^ 168.994 and 18.63 mg/L for *m/z* ^−^ 268.980. The LODs of representative key peaks determined using fitting results (Supplementary Figures S1, S2) and the minimal concentrations used in SIMS analysis, aka LOQs were listed in Supplementary Table S2. Specifically, the LOQs are 2.50 mg/L for both *m/z* ^−^ 168.994 and *m/z* ^−^ 268.980 based on experimental values.

The LODs can be improved by increasing the secondary ion yields via either wider pulse width or longer analysis frames. Recent results have shown at least an order of magnitude increase in LODs using this approach (Coullerez et al., 2003; Klump et al., 2018). Sample properties, such as cohesive energy and density, could influence the LOD or surface sensitivity of the primary ion source due to the amount of surface erosion (Muramoto et al., 2012). Method development and optimization is needed to improve the LOD of the target PFAS analytes using ToF-SIMS in the future.

The estimated LODs does not seem to be low as what the more developed LC-MS/MS methods could offer. However, real field water sample analysis results, to be discussed below in more details, show that the SIMS LODs are equivalent to the ppt level LODs from LC-MS/MS. The surface sensitivity of ToF-SIMS has been widely used in forensic analysis (Lee et al., 2016; Cai et al., 2017; Terlier et al., 2020), such sensitivity is not easily translatable to an equivalent LODs in terms of the conventional definition of bulk samples. Regardless, because the forensic surface analysis capabilities inherent of ToF-SIMS, trace PFASs can be detected using simple sample preparation and a small amount (i.e., microliter) of water samples.

### 3.2 Repeatability and relative mass accuracy of PFAS peak detection

During SIMS spectral analysis, relative mass accuracy, defined as Δm = Abs (10^6^ × (m/z^—^_obs_ − m/z^—^_the_)/m/z^—^_the_) in ppm, and measurement repeatability shown as standard deviation (S.D.) are key factors for obtaining reliable peak identifications. Peak identifications of analyzed PFAS compounds are summarized in Table 1 and Supplementary Table S2. The relative standard deviation (RSD%) is calculated as peak area S.D. divided by the mean peak area. The RSD% results of representative peaks based on the PFBA and PFOS samples are listed in Supplementary Tables S3–S5, respectively. The RSDs% are generally less than 2.5% for PFOS and PFBA, indicating good reproducibility.

**TABLE 1:** Possible peak assignment of PFOS and PFBA using ToF-SIMS in the negative mode.

*m/z* ^−^_obs_ ^a^	*m/z* ^−^_the_ ^b^	Δm^c^ (ppm)	Suggested formula	References
68.999	68.995	3.70	CF_3_^−^	Berger et al. (2004)
98.956	98.955	20.11	FSO_3_^−^	Llorca et al. (2009), Mulabagal et al. (2018)
118.987	118.992	18.31	C_2_F_5_^−^	Berger et al. (2004)
129.954	129.954	18.67	CF_2_SO_3_^−^	Berger et al. (2004)
168.994	168.989	19.34	C_3_F_7_^−^	Llorca et al. (2009)
179.951	179.950	16.52	C_2_F_4_SO_3_^−^	Berger et al. (2004)
212.968	212.979	10.79	C_4_F_7_O_2_^−^	Navarro et al. (2011), Mulabagal et al. (2018)
218.986	218.986	10.03	C_4_F_9_^−^	Llorca et al. (2009)
229.949	229.947	11.18	C_3_F_6_SO_3_^−^	Berger et al. (2004)
268.980	268.982	3.37	C_5_F_11_^−^	Langlois et al. (2007)
318.962	318.979	5.34	C_6_F_13_^−^	Navarro et al. (2011), Mulabagal et al. (2018)
362.937	362.969	18.49	C_7_F_13_O_2_^−^	Navarro et al. (2011), Mulabagal et al. (2018)
368.833	368.976	17.01	C_7_F_15_^−^	Berger et al. (2004)
398.915	398.936	12.44	C_6_F_13_SO_3_^−^	Chan et al. (2009)
418.964	418.973	9.01	C_8_F_17_^−^	Berger et al. (2004), Berger and Haukås (2005), Navarro et al. (2011), Mulabagal et al. (2018)
429.937	429.934	19.94	C_7_F_14_SO_3_^−^	Berger et al. (2004)
462.942	462.963	2.46	C_9_F_17_O_2_^−^	Navarro et al. (2011), Mulabagal et al. (2018)
498.914	498.930	32.07	C_8_F_17_SO_3_^−^	Berger et al. (2004), Berger and Haukås (2005), Mulabagal et al. (2018)

^a^ *m/z* ^−^_obs_: observed mass to charge ratio in the negative ion mode.

^b^ *m/z* ^−^_the_: theoretical mass to charge ratio in the negative ion mode.

^c^ Δm: = Abs (10^6^ × (*m/z* ^−^_obs_-*m/z* ^−^_the_)/*m/z* ^−^_the_) (expressed in ppm) (Gilmore and Seah, 2000).

The values of relative mass accuracy of most peaks are less than 30 ppm in the negative mode and less than 100 ppm in the positive mode, suggesting that the peak identification is dependable. The standard deviations of most peak areas are between 10% and 20% among all parallel samples. The standard deviations of peak height are larger than those of peak areas. Using peak area for peak identification would be more dependable in measurement evaluation because the intensity is spread over the mass scale due to imperfect energy compensation and topography effects for a specific ion. Therefore, the peak height consequently gives a smaller value with poorer repeatability. Thus, the peak area standard deviation is better to describe SIMS spectral repeatability. The PFAS measurement repeatability results shown in this work are satisfactory for static ToF-SIMS as a semi-quantitative analysis technique (Gilmore and Seah, 2000; Gilmore et al., 2005; Gilmore et al., 2007). Most importantly, SIMS offers sensitive and reproducible detection of characteristic ions and ion fragments of PFAS readily as shown in Supplementary Figures S3, S4.

### 3.3 Representative PFAS molecular identification

**FIGURE 1:**
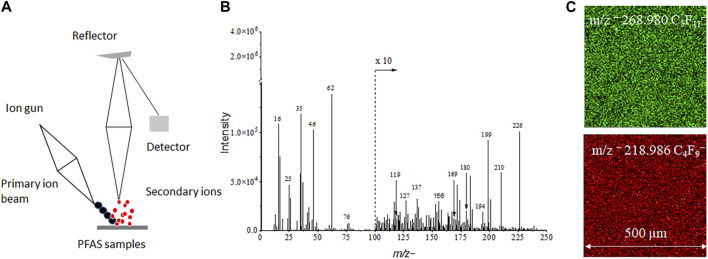
**(A)** A ToF-SIMS schematic showing PFAS analysis in mass spectral **(B)** and 2D imaging **(C)** mode.

The schematic of spectral and 2D image analysis using ToF-SIMS is depicted in Figure 1. The volume of PFAS reference and groundwater samples is 25 μL and the sample can be easily prepared followed with analysis in ToF-SIMS without additional sample treatment. This method is simple in comparison with other techniques such as GC-MS/MS and LC-MS/MS. The latter requires extraction with organic solvents or pretreatment before analysis (Bach et al., 2016; Dauchy, 2019). ToF-SIMS is a mass spectral imaging technique, and both spectra and images are acquired during measurements. Characteristic peaks for each PFAS reference materials are observed in ToF-SIMS mass spectra (See Table 1 and Supplementary Table S2). Moreover, 2D images give direct visualization of the distribution of PFAS components, like the *m/z* ^−^ 268.980 C_5_F_11_^−^ and *m/z* ^−^ 218.986 C_4_F_9_^−^ in a mixture. This feature of spatial distribution of different components is especially appealing in studying complex PFAS mixtures.

**FIGURE 2:**
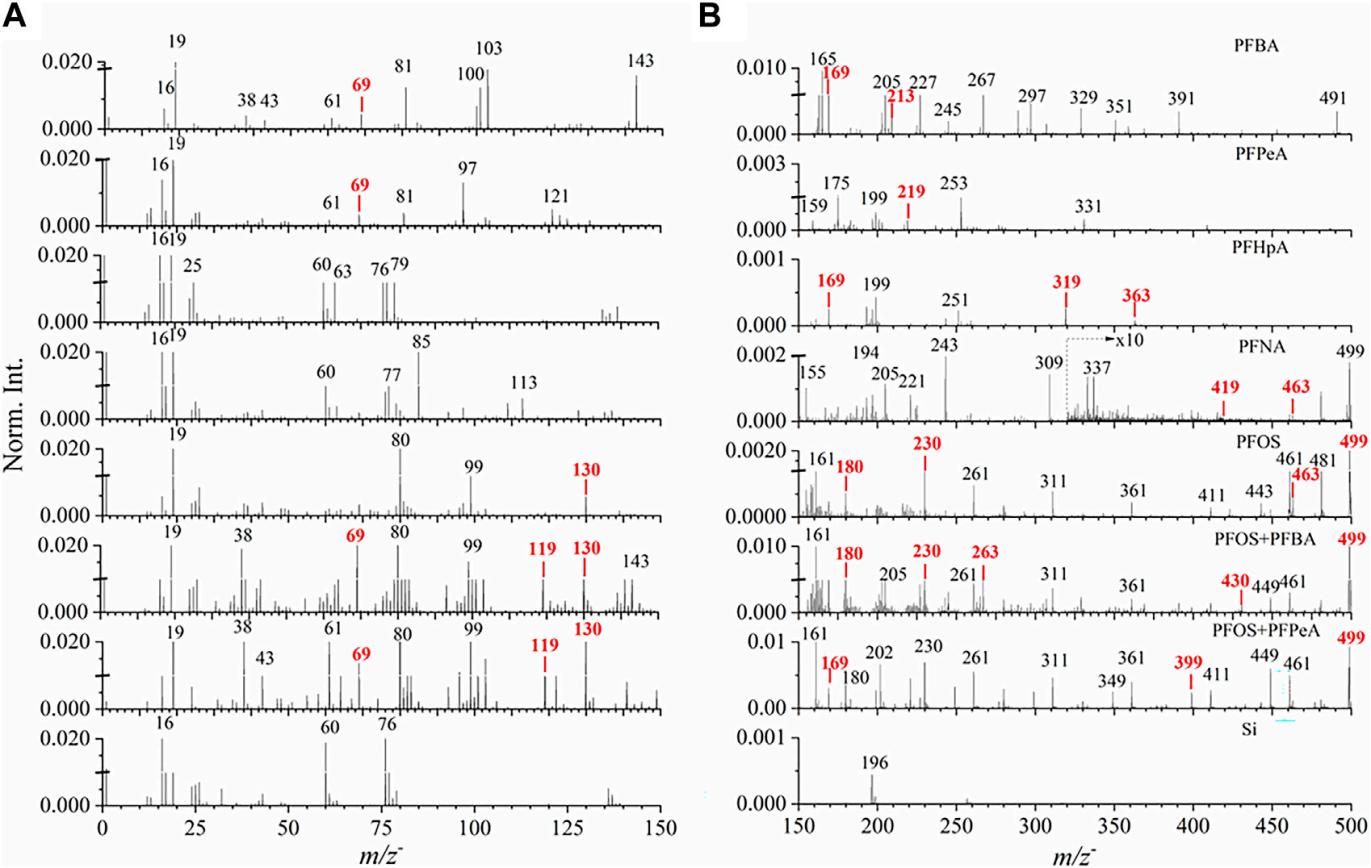
ToF−SIMS spectral comparison of seven reference PFASs samples and clean Si wafer in the mass range of **(A)** *m/z* ^−^ 0–150 and **(B)** *m/z* ^−^ 150–500 in the negative ion mode. Red color mark indicates main fragment ion peaks and pseudo-molecular peaks in each spectrum. Norm. Int. stands for Normalized Intensity. The peaks are marked in integers for visual convenience. More details are seen in Table 1 and Supplementary Table S2.

To assure the precision of SIMS spectral measurements, at least five repetitions were acquired for every PFAS reference material and real-world samples in the positive and negative mode, respectively. Good repeatability is illustrated in Supplementary Figures S3, S4. Figure 2 depicts ToF-SIMS spectral comparison of long-chain and short-chain PFASs, including PFBA, PFPeA, PFHpA, PFNA, PFOS, two mixtures containing PFBA and PFOS as well as PFPeA and PFOS, and the Si wafer control in the mass range of *m/z* ^−^ 0–500 in the negative mode. Additionally, the positive spectral comparisons are depicted in Supplementary Figure S5.

Three main spectral results are shown in Figure 2. First, PFASs, including several representative PFCA compounds and PFOS, are fluorinated compounds. The fluoride ion peak, *m/z* ^−^ 18.999 F^−^, was observed with much higher intensities in all samples, indicating the detection of fluorine fragments. This finding shows the convenience of direct fluoride detection using SIMS compared to other bulk conversion methods (McDonough et al., 2019; Schultes et al., 2019). Second, typical pseudo-molecular ions [M-H]^−^ peaks were observed and identified for each PFAS reference compound in the negative mode (Table 1). Specifically, these ions are *m/z* ^−^ 212.968 C_4_F_7_O_2_^−^ for PFBA, 262.894 C_5_F_9_O_2_^−^ for PFPeA, 362.937 C_7_F_13_O_2_^−^ for PFHpA, 462.942 C_9_F_17_O_2_^−^ for PFNA, and 498.914 C_8_F_17_SO_3_^−^ for PFOS (Figure 2), respectively. As to the two mixtures consisting of PFBA and PFOS and PFPeA and PFOS, the corresponding molecular ions peaks (i.e., *m/z* ^−^ 212.968 C_4_F_7_O_2_^−^ and 498.914 C_8_F_17_SO_3_^−^; 262.894 C_5_F_9_O_2_^−^ and 498.914 C_8_F_17_SO_3_^−^) are observed in Figure 2B, respectively.

Additionally, characteristic PFASs fragment ion peaks were observed and identified (Table 1). Figure 2A shows that the main fragment ion peaks for PFBA are *m/z* ^−^ 68.999 CF_3_^−^, 118.987 C_2_F_5_^−^, and 168.994 C_3_F_7_. Some higher intensity mass peaks (i.e., *m/z* ^−^ 81.020, 99.983, 169.034) belong to the fragments of PFBA according to the NIST WebBook reference mass spectra (Linstrom, 1997; NIST, 2023). However, they cannot be identified according to present literature. The pseudo-molecular peak *m/z* ^−^ 212.979 of PFBA is evident in the spectrum. PFPeA, PFHpA, and PFNA share common fragments peaks with PFBA, including *m/z* ^−^ 68.999 CF_3_^−^, 118.987 C_2_F_5_^−^, and 168.994 C_3_F_7_^−^, because of similar molecular structures. In contrast, some peaks show relatively lower intensity possibly due to fragmentation difference among compounds. With the increase of molecular weight of reference PFAS chemicals, a series of fragments were observed, such as *m/z* ^−^ 218.986 C_4_F_9_^−^ (Figure 2B), 318.962 C_6_F_13_^−^ (Figure 2B), and 368.883 C_7_F_15_^−^ (Figure 2B). Similarly, some unidentified peaks (e.g., *m/z* ^−^ 81.020, 96.979, 120.952) are representative in PFPeA fragments. Peaks, such as *m/z* ^−^ 61.001, 76.974, 85.001, 112.992, 155.016, 220.933, and 242.943, come from PFNA fragments according to the NIST WebBook (Linstrom, 1997).

From the SIMS spectral comparison, representative fragment ion peaks from PFOS were observed and identified in Figure 2, such as *m/z* ^−^ 79.969 SO_3_^−^, 98.956 FSO_3_^−^, 129.954 CF_2_SO_3_^−^, 179.951 C_2_F_4_SO_3_^−^, and 229.949 C_3_F_6_SO_3_^−^. In the lab-prepared mixture samples, these peaks have significant occurrences with higher mass counts due to the presence of PFOS. Previous analyses using HPLC-MS/MS also report these characteristic peaks from PFOS (Berger et al., 2004). Higher intensity peaks, such as *m/z* ^−^ 310.954, 361.023, and 460.923, without identification might be related to PFOS, because these peaks appear in the spectra of PFOS and the two-component mixtures containing PFOS. The signal to noise ratios (SNRs) for the labeled ions with low relative abundance in the spectra, such as *m/z* ^*−*^ 118.987, 212.986, 268.980, 362.937, 368.833, and 419.984, are 3970, 124, 206, 71, 323 and 27, respectively, which indicate that these ions exist in the PFAS samples with reasonable signal intensities.

### 3.4 PFAS mixture chemical spatial distribution

Spectral PCA was conducted to confirm the observation of spectral analysis of representative two-component mixtures, including PFBA and PFOS and PFPeA and PFOS, respectively, and to further elucidate characteristic PFAS peaks. Figs. S6a − S6b depict the scores plots of principal component one (PC1), PC2, and PC3; and Figs. S6c − S6d give the corresponding loadings plots in the negative ion mode. Representative pseudo−molecular ion peaks of PFBA, PFPeA, and PFOS have high loadings in the loadings plots. They act as key contributors separating selected PFASs as expected. For example, PFBA, PFHpA, and PFNA are situated in the PC1 positive score quadrant (Supplementary Figure S6A), suggesting that their molecular peaks should have positive PC1 loadings. The PCA results also demonstrate that molecular peaks, such as *m/z* ^−^ 212.968 C_4_F_7_O_2_^−^, 362.937 C_7_F_13_O_2_^−^ and 462.942 C_9_F_17_O_2_^−^ corresponding to PFBA, PFHpA, and PFNA in positive PC1 loadings, respectively, are main contributors in the separation among different samples (Supplementary Figure S6C). PC1 cannot separate PFPeA from other samples, while PC2 and PC3 can with PFPeA residing in the PC2 positive scores plot (Supplementary Figure S6A) and PC3 negative scores plot (Supplementary Figure S6B). The molecular peak of PFPeA *m/z* ^−^ 262.894 C_5_F_9_O_2_^−^ is situated in PC2 positive (Supplementary Figure S6D) and PC3 negative loading plots (Supplementary Figure S6E). The mixture substances and PFOS are only separated from other samples by PC1 due to the common component PFOS, and PC1 loadings plots (Supplementary Figure S6C), and the molecular peak of *m/z* ^−^ 498.914 C_8_F_17_SO_3_^−^ is situated in PC1 negative.

**FIGURE 3:**
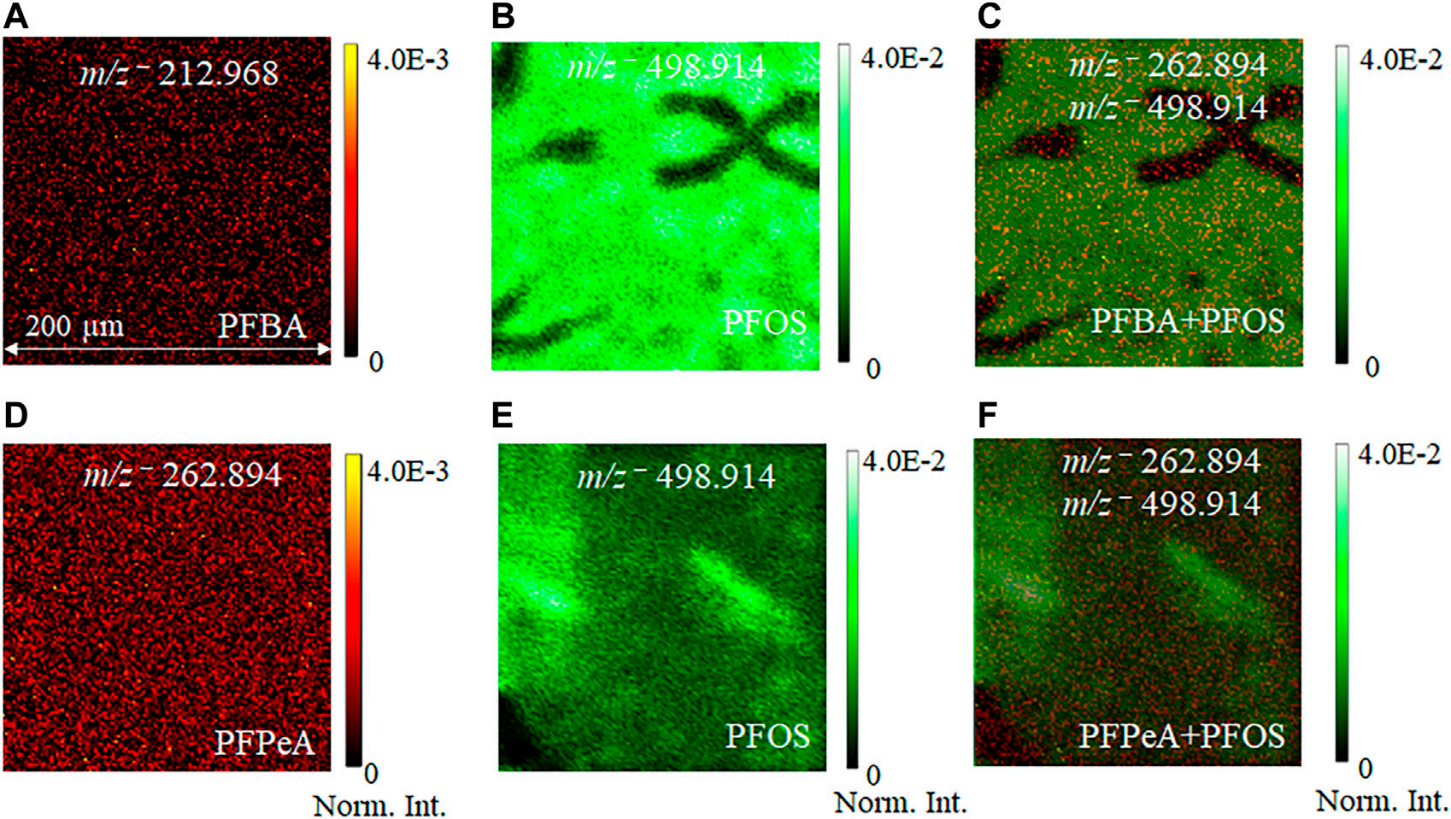
Comparison of normalized 2D ToF-SIMS images of pseudo−molecular ion distributions from the single component PFAS sample and the two−component mixture: **(A)** PFBA *m/z* ^−^ 212.968, **(B)** PFOS *m/z* ^−^ 498.914, **(C)** PFBA + PFOS *m/z* ^−^ 212.968, 498.914; **(D)** PFPeA *m/z* ^−^ 262.894; **(E)** PFOS *m/z* ^−^ 498.914; and **(F)** PFPeA + PFOS *m/z* ^−^ 262.942 and 498.914, respectively. 2D images are normalized to the total ion counts for ease of comparisons.

Figure 3 depicts the normalized 2D image comparison of the spatial distribution of molecular ion peaks among the single components and mixtures in the negative ion mode. The dark sub-regions in Figures 3B,C indicate low ion counts. Detection of PFAS mixture compounds without further sample treatment shows selectivity of ToF-SIMS as a technique to analyze PFASs. Figures 3A−C represents the 2D normalized images and distributions of key peaks of the single components and the two-component mixture consisting of PFOS and PFBA. Representative molecular ion peaks of *m/z* ^−^ 212.968 PFBA are in red and *m/z* ^−^ 498.914 PFOS in green. PFOS shows a higher molecular ion peak intensity than PFBA. It is not surprising that the PFOS is predominant in the mixture, in agreement with findings in the spectral analysis. Similarly, the second mixture of PFPeA and PFOS (Figures 3D−F) shows consistent results as the other mixture in Figures 3A–C. The normalized intensity of the molecular ion peak *m/z* ^−^ 498.914 PFOS is higher than that of *m/z* ^−^ 262.894 PFPeA. 2D SIMS images give direct visualization of main components as an attractive feature in mass spectral imaging, showing long-chain and short-chain PFASs spatial distribution. This is a unique SIMS feature that bulk MS analysis could not provide.

### 3.5 Sensitive detection of PFAS in real−world groundwater

**FIGURE 4:**
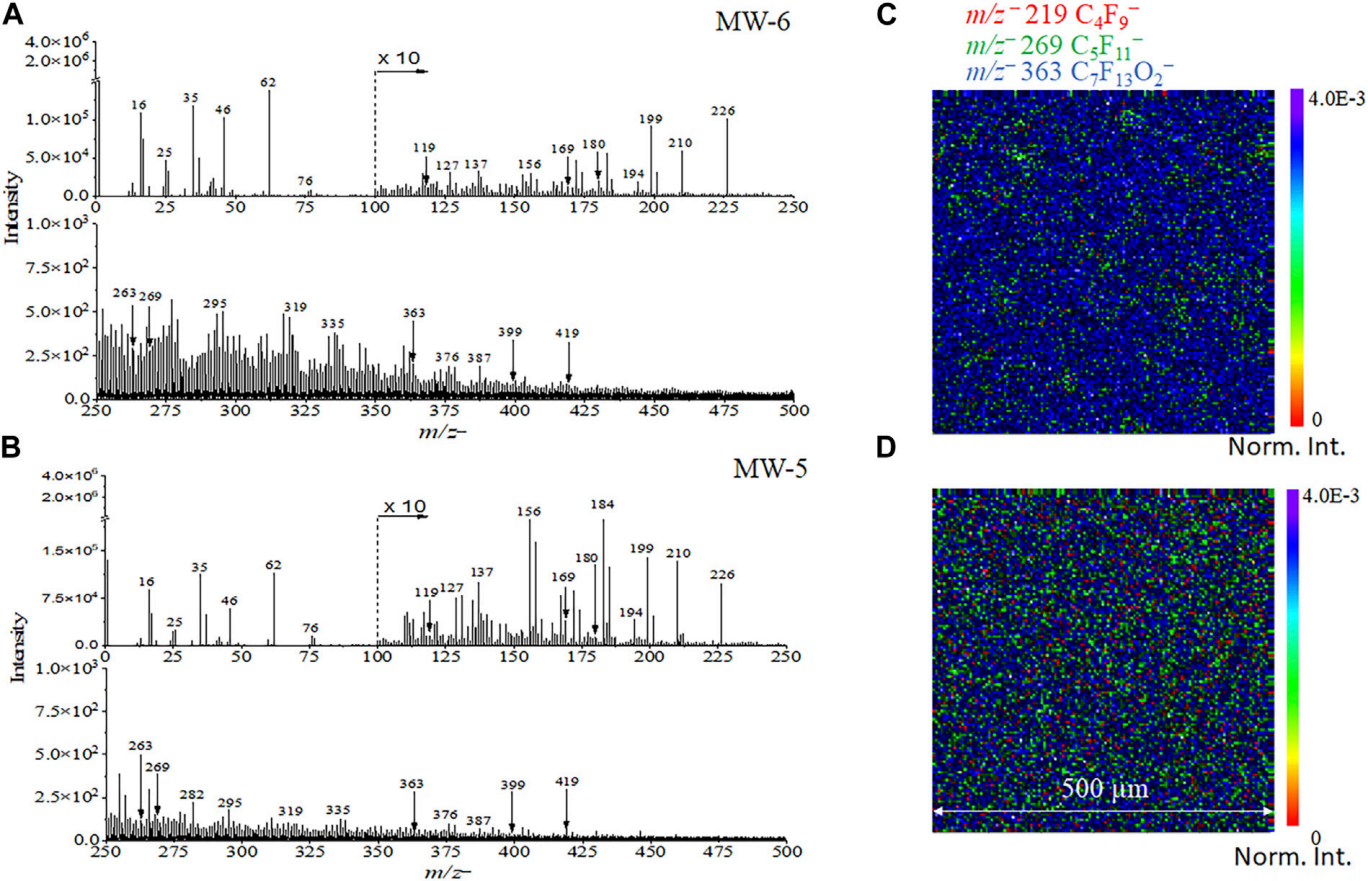
ToF−SIMS spectral results of **(A)** MW-6 and **(B)** MW-5 real world groundwater samples from the field. Normalized 2D SIMS images of selected ions of MW-6 **(C)** and MW-5 **(D)** containing PFASs. 2D images are normalized to the total ion counts for ease of comparisons.

Figure 4A−b show the spectral comparison plots of real groundwater samples. Fluorinated compounds, such as *m/z* ^−^ 168.994 C_3_F^−^, 268.980 C_5_F_11_^−^, and 368.833 C_7_F_15_^−^, can be detected in two groundwater samples named MW-5 and MW-6, respectively. In addition, characteristic pseudo-molecular ions [M-H]^−^ peaks were observed and identified for the groundwater samples in the negative mode, such as *m/z* ^−^ 262.894 C_5_F_9_O_2_^−^ and *m/z* ^−^ 362.937 C_7_F_13_O_2_^−^. Furthermore, fragment ion peaks with relatively higher masses from PFOS, like *m/z* ^−^ 179.951 C_2_F_4_SO_3_^−^, were observed in groundwater samples. This finding indicates that ToF-SIMS is an extremely sensitive technique for the PFPeA and PFOS detection from the environmental water sample. Interestingly, the representative normalized 2D images of the PFASs related peaks, including *m/z* ^−^ 218.986 C_4_F_9_^−^, *m/z* ^−^ 268.980 C_5_F_11_^−^, *m/z* ^−^ 318.962 C_6_F_13_^−^, and *m/z* ^−^ 368.833 C_7_F_15_^−^, were observed (Figure 4C), giving direct evidence of PFAS detection. The polluted ground water containing PFAS was in the form of a slurry. Dilution was used to dissipate the particles more evenly on the Si substrate. The mass ion spatial distribution depicted in Figure 4 gives a representation of ions of interest in the complex mixture and their relative abundance to each other in a small volume, namely, several microliters were used to prepare the sample. The relative abundances of the selected ions are different between MW5 and MW6, which were collected from different wells in a polluted site.

Supplementary Figure S8 depicts the comparisons of SIMS 2D images of *m/z* ^*−*^ 219, 269, and 363 between the Si substrate (a–c) and the ground water sample MW-6 (d–f), respectively. Unlike the 2D normalized images in Figure 3, 4 in the main text, these results are shown in the measurement counts. The counts of the real-world sample MW-6 are on the order of 10^4^ for peaks of interest. Such intensity indicates that the detected peaks are real and not noise. Comparable results of MW-5 are depicted in Supplementary Figure S9. Spatial distribution of PFAS is important because there is a huge interest to understand the PFAS laden materials to address grand challenges in understanding the fate PFAS degradation and environmental restoration. First, having the PFAS distribution will help answer the question of where PFAS compounds reside in the PFAS-laden materials, e.g., clay or resin as amendment. Liquid extraction can tell you the amount of PFAS but not the location. Also, the ability to offer chemical maps of PFAS and its PFAS degradation products in the PFAS loaded amendments would be attractive to decipher the reaction pathways. Again, liquid extraction and bulk LC-MS or GC-MS analyses could tell you how much not where and how relative to the original location of the PFAS.

**FIGURE 5:**
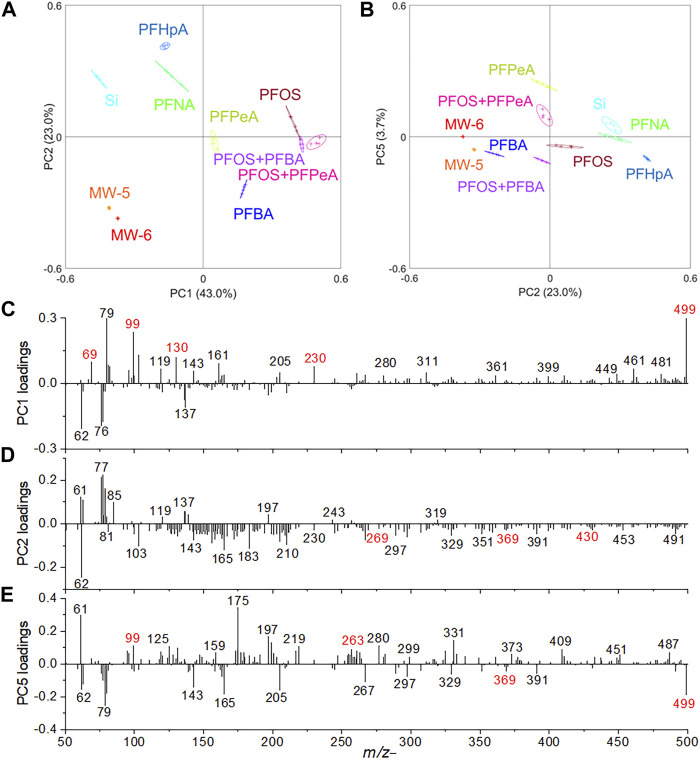
Selected peak spectral PCA results in the negative mode: **(A)** scores plot of PC1 vs. PC2, **(B)** scores plot of PC2 vs. PC5, **(C)** PC1, **(D)** PC2, and **(E)** PC5 loadings plots.

Selected peak spectral PCA was conducted to confirm the observation of spectral analysis of laboratory prepared mixtures and real-world groundwater samples. As shown in Figure 5A, PC1 and PC2 can explain more than 66% of all data. PFPeA, PFBA, PFOS, mixture of PFOS and PFBA, and mixture of PFOS with PFPeA are situated in the PC1 positive score quadrant, suggesting the molecular peaks have high positive PC1 loadings, including *m/z* ^−^ 68.999 CF_3_^−^, *m/z* ^−^ 98.956 FSO_3_^−^, *m/z* ^−^ 129.954 CF_2_SO_3_^−^, *m/z* ^−^ 229.949 C_3_F_6_SO_3_^−^, and *m/z* ^−^ 498.914 C_8_F_17_SO_3_^−^. PC2 separates the PFHpA, PFNA, and PFOS from the two groundwater samples (i.e., MW-5 and MW-6), PFPeA, PFBA, PFOS + PFBA, and PFOS + PFPeA. The characteristic peaks in the PC2 negative loadings are *m/z* ^−^ 268.980 C_5_F_11_^−^, *m/z* ^−^ 368.833 C_7_F_15_^−^, and *m/z* ^−^ 429.937 C_7_F_14_SO_3_^−^. This finding is consistent with the spectral analysis results as discussed before. In addition, Figure 5B shows the PCA results of PC2 vs. PC5. PC5 separates the two groundwater samples containing PFASs, and the relevant characteristic peaks are shown in the loadings plots, for example, the peak *m/z* ^−^ 262.894 C_5_F_9_O_2_^−^ has a higher loading in PC5 positive, and *m/z* ^−^ 362.937 C_7_F_13_O_2_^−^ has a higher loading in PC5 negative. Loadings plots of PC1, PC2, and PC5 are shown in Figures 5C–E respectively.

The ppt level concentrations of approximately 20 ppb of PFASs in the field groundwater samples were verified based on the commercial laboratory analysis using LC-MS/MS. Additional comparison and quantification will be investigated in the next step. Thus, our results demonstrate that ToF-SIMS can detect PFAS at concentrations in ppt level using micrometer of real water samples, i.e., significantly lower than the estimated LODs. Furthermore, our finding show that ToF-SIMS has the potential to tackle with the challenge of determining PFAS contamination in drinking water and groundwater using the forensic analysis (Zhou et al., 2016; Terlier et al., 2020) and source tracking capabilities (Kempson et al., 2003; Héberger, 2008; Kind and Fiehn, 2010). The latter is a topic that is worth of additional investigation.

PFAS contamination in groundwater and soil is a major concern in the environment (Nzeribe et al., 2019; Anderson et al., 2021; Kurwadkar et al., 2022). Concerns over investigation-derived waste (IDW) continues to grow (Singh et al., 2019; Lenka et al., 2021; Longendyke et al., 2022). IDW refers to water, soil and drill cuttings produced during well installations and sampling activities performed during contaminated site investigations. Recently, the memorandum of Temporary Prohibition on Incineration of Materials Containing PFASs calls for a better understanding of PFAS-laden materials (DOD, 2022). Our results show that ToF-SIMS can provide mass spectra in one-dimension and 2D maps of PFAS as well as PFAS dissociation products. Therefore, ToF-SIMS, as an imaging technique, offers a unique and much needed solution to analyzing and imaging PFAS compounds directly on the surface or substrate of the PFAS laden materials, unlike the bulk LC-MS/MS or GC-MS/MS approaches. The latter methods require sample preparation and extraction, which destroys the PFAS-laden materials.

## 4 Conclusion

In conclusion, we demonstrate that ToF-SIMS can be used to analyze persistent PFAS pollutants with simple sample preparation due to its superior surface sensitivity. Characteristic pseudo-molecular ion peaks of several representative PFASs were observed. In both simulated mixture samples and real groundwater samples, 2D visualization of PFASs, including PFOS, component distributions are possible. Overall, our results show that ToF-SIMS is viable to detect PFASs in groundwater using a minute amount of liquid sample with easy sample preparation. SIMS as a mass spectral imaging technique is attractive due to its simplicity in sample preparation, small volume of samples, and efficiency of sample analysis. More importantly, the forensic potential of ToF-SIMS in detecting trace amount of PFASs in wastewater is appealing in understanding PFAS contamination in drinking water and groundwater and pollutant source tracking. More environmental water samples are warranted for analysis in ToF-SIMS to provide a rich reference library of data for its future applications to better determine PFASs in the environmental water.

## Data Availability

The original contributions presented in the study are included in the article/Supplementary Material, further inquiries can be directed to the corresponding author.
