# Supplementary material for: Molecular detection of per- and polyfluoroalkyl substances in water using time-of-flight secondary ion mass spectrometry
Source: Front Chem. 2023 Oct 6;11:1253685. doi: 10.3389/fchem.2023.1253685 (PMC10587417; doi:10.3389/fchem.2023.1253685)
Supplement: Supplementary file 1 [file DataSheet1.docx]

Supplementary Material

Molecular Detection of Per- and Polyfluoroalkyl Substances in Water Using Time-of-Flight Secondary Ion Mass Spectrometry

Xiao-Ying Yu ^1*^, Cuiyun Yang ^1, †^, Jun Gao ^1, †^, Zhong (John) Xiong ^2^, Xiao Sui ^3^, Lirong Zhong ^4^, Yuchen Zhang ^1^, and Jiyoung Son ^4^

^1^Materials Science and Technology Division, Oak Ridge National Laboratory, Oak Ridge, TN 37830, USA

^2^Haley & Aldrich, Inc., 3187 Red Hill Avenue, Suite 155, Costa Mesa, California, 92626, USA

^3^College of Geography and Environment, Shandong Normal University, Jinan 250358, China

^4^Energy and Environment Directorate, Pacific Northwest National Laboratory, Richland, WA 99354, USA

^†^These authors contributed equally.

*** Correspondence:**Dr. Xiao-Ying Yu
yuxiaoying@ornl.gov

Table of Contents

[1 Supplementary Figures 4](#_Toc146016184)

[Figure S1. (a) The relationship between molecular signal intensities *m/z*^−^ 168.994 and concentrations of PFPeA. (b) Four concentrations are 0.002%, 0.001%, 0.005%, 0.0025%. The dash line is the linear least-squares fit. 4](#_Toc146016185)

[Figure S2. (a) The relationship between molecular signal intensities of *m/z*^−^ 268.980 and concentrations of PFOS. (b) Three concentrations are 0.001%, 0.005%, and 0.0025%. The dash line is the linear least-squares fit. 5](#_Toc146016187)

[Figure S3a. Reproducibility of PFBA SIMS spectral analysis in the range of *m/z*^−^ 0 – 200 in the negative mode. 6](#_Toc146016188)

[Figure S3b. Reproducibility of PFBA SIMS spectral analysis in the range of *m/z*^−^ 200 – 500 in the negative mode. 7](#_Toc146016189)

[Figure S4a. Reproducibility of PFBA SIMS spectral analysis in the range of *m/z*^+^ 0 – 200 in the positive mode. 8](#_Toc146016190)

[Figure S4b. Reproducibility of PFBA SIMS spectral analysis in the range of *m/z*^+^ 200 – 500 in the positive mode. 9](#_Toc146016191)

[Figure S5a. SIMS spectral comparison of representative PFASs in the range of *m/z*^+^ 0 – 150 in the positive mode. 10](#_Toc146016192)

[Figure S5b. SIMS spectral comparison of representative PFASs in the range of *m/z*^+^ 150 – 500 in the positive mode. 11](#_Toc146016193)

[Figure S6. Spectral PCA results of selected peaks in the negative mode: (a) PC1 vs. PC2 scores plot; (b) PC1 vs. PC3 scores plot; (c) PC1; (d) PC2; and (e) PC3 loadings plots. 12](#_Toc146016194)

[Figure S7. All peak spectral PCA results of PFOA and PFOS compounds in the positive mode: (a) PC1 vs. PC2 scores plot; (b) PC1 and (c) PC2 loadings plots. 14](#_Toc146016195)

[Figure S8. Comparisons of SIMS 2D images of *m/z^−^* 219, 269, and 363 between the Si wafer substrate (a – c) and the deposited MW-6 ground water sample (d – f), respectively in the negative ion mode. 15](#_Toc146016196)

[Figure S9. SIMS 2D images of *m/z^−^* 119 (a), 213 (b), 263 (c), 363 (d), 269 (e), and 419 (f) from the ground water sample MW-5 in the negative ion mode. 16](#_Toc146016197)

[2 Supplementary Tables 17](#_Toc146016198)

[Table S1. Summary descriptions of representative PFAS reference and mixture samples. 17](#_Toc146016199)

[Table S2. LODs and LOQs of selected peaks of PFAS compounds. 18](#_Toc146016200)

[Table S3. Possible peak assignment of PFOS and PFBA using ToF-SIMS in the positive mode. 19](#_Toc146016201)

[Table S4. Static ToF-SIMS measurement repeatability of PFBA representative peaks in the positive and negative mode. 20](#_Toc146016202)

[Table S5. Static ToF-SIMS measurement repeatability of PFOS representative peaks in the positive and negative mode. 21](#_Toc146016203)

# Supplementary Figures


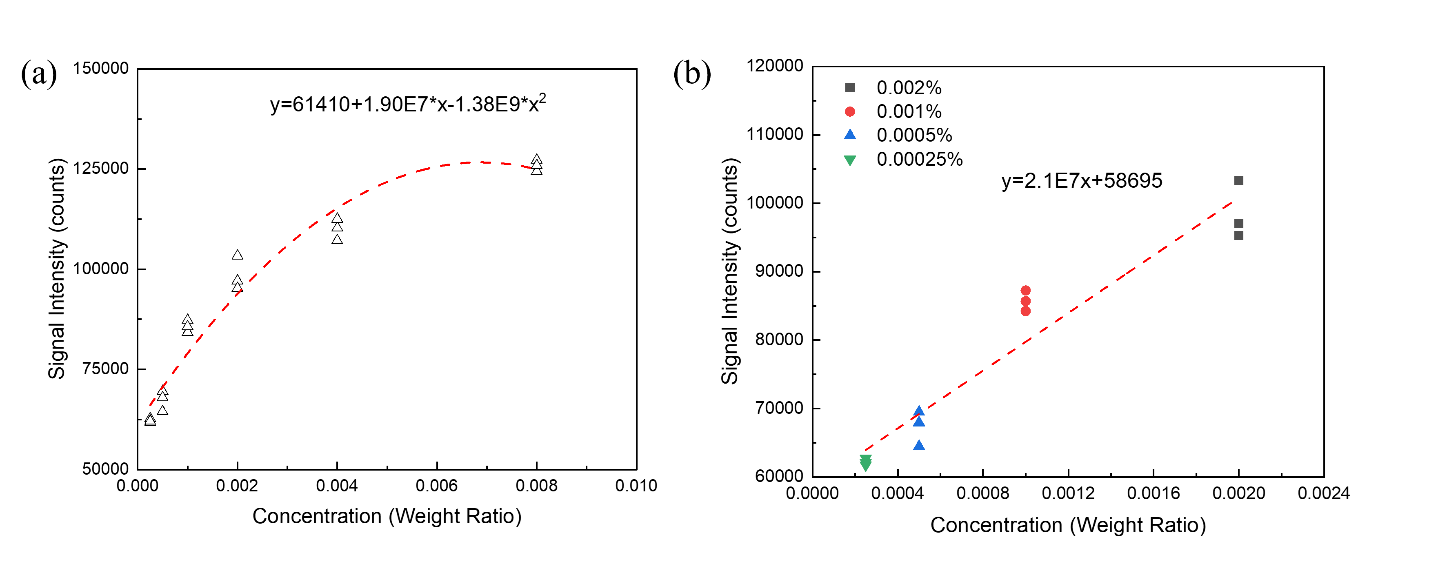


## **Figure S1.** (a) The relationship between molecular signal intensities *m/z*^−^ 168.994 and concentrations of PFPeA. (b) Four concentrations are 0.002%, 0.001%, 0.005%, 0.0025%. The dash line is the linear least-squares fit.

The R-square is 0.95 for the fitting in Figure S1 (a), and the adjusted R-square is 0.92 for the fitting in Figure S1(b). The weigh ratio is calculated as the following, 1 mg PFPeA diluted in 100g water, the concentration is 0.001%. It is equivalent to 0.01 mg/mL or 0.1 mg/L.


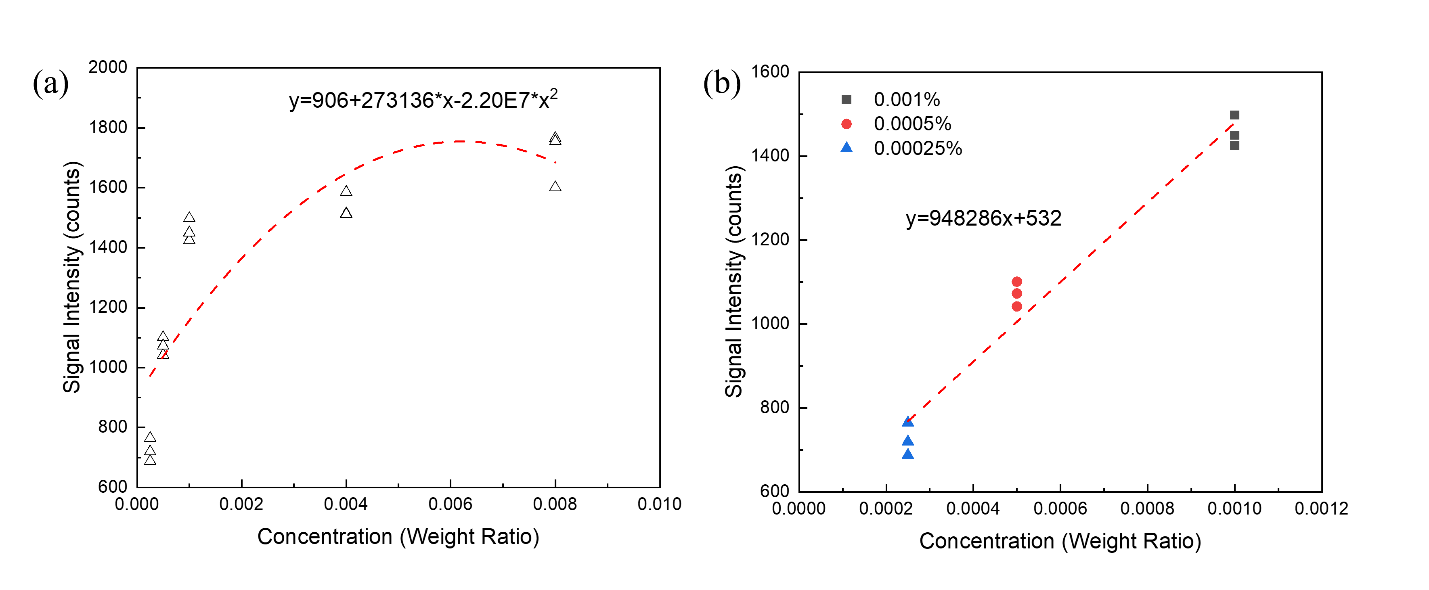


## **Figure S2.** (a) The relationship between molecular signal intensities of *m/z*^−^ 268.980 and concentrations of PFOS. (b) Three concentrations are 0.001%, 0.005%, and 0.0025%. The dash line is the linear least-squares fit.

**Figure S2** illustrates that the supported experimental data and that the linear least-squares fit can be used to determine the LOD of PFOS using a series of solutions deposited on Si wafers in static ToF-SIMS. The R-square is 0.68 for the fitting in Figure S2a, and the R-square for the fitting is 0.96 in Figure S2b.

**
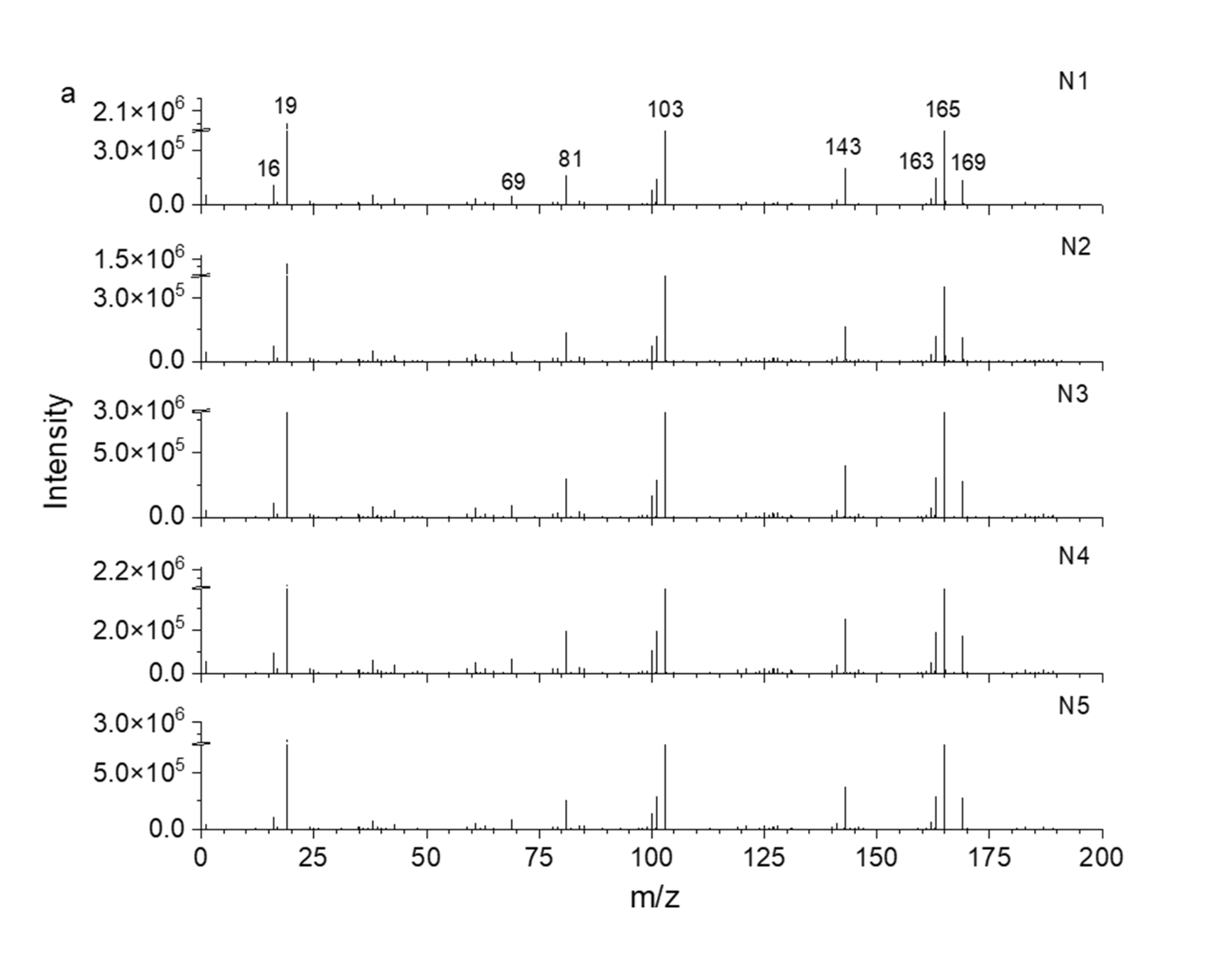
**

## Figure S3a. Reproducibility of PFBA SIMS spectral analysis in the range of *m/z*^−^ 0 – 200 in the negative mode.

N1, 2, 3, 4, and 5 represent five consecutive measurements of PFBA in the negative ion mode. ToF-SIMS spectral measurements show good reproducibility. Repeatability results are summarized in **Table 3** in the main text.


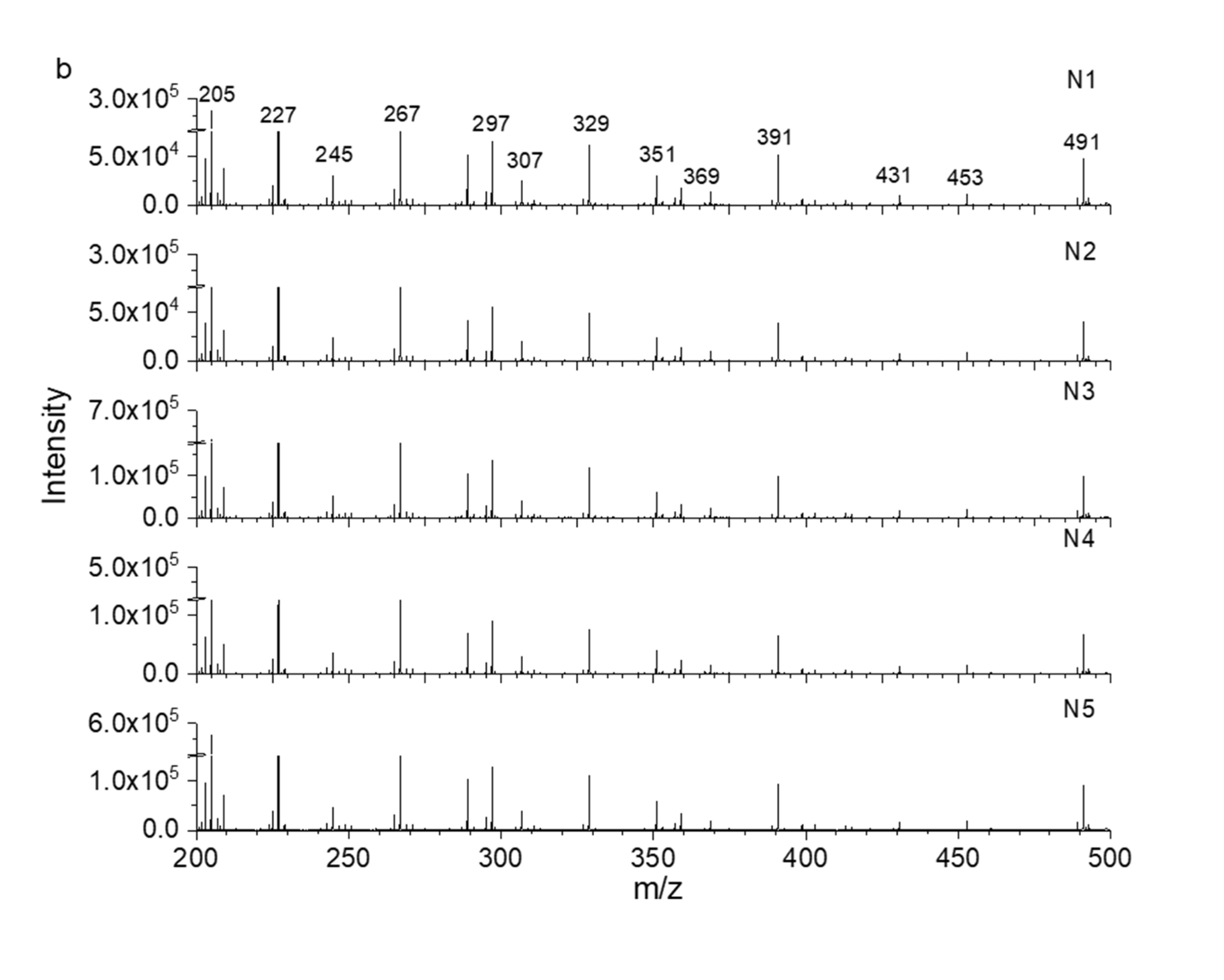


## Figure S3b. Reproducibility of PFBA SIMS spectral analysis in the range of *m/z*^−^ 200 – 500 in the negative mode.

N1, 2, 3, 4, and 5 represent five consecutive measurements of PFBA in the negative mode. ToF-SIMS spectral measurements show good reproducibility. Repeatability results are summarized in **Table 3** in the main text.


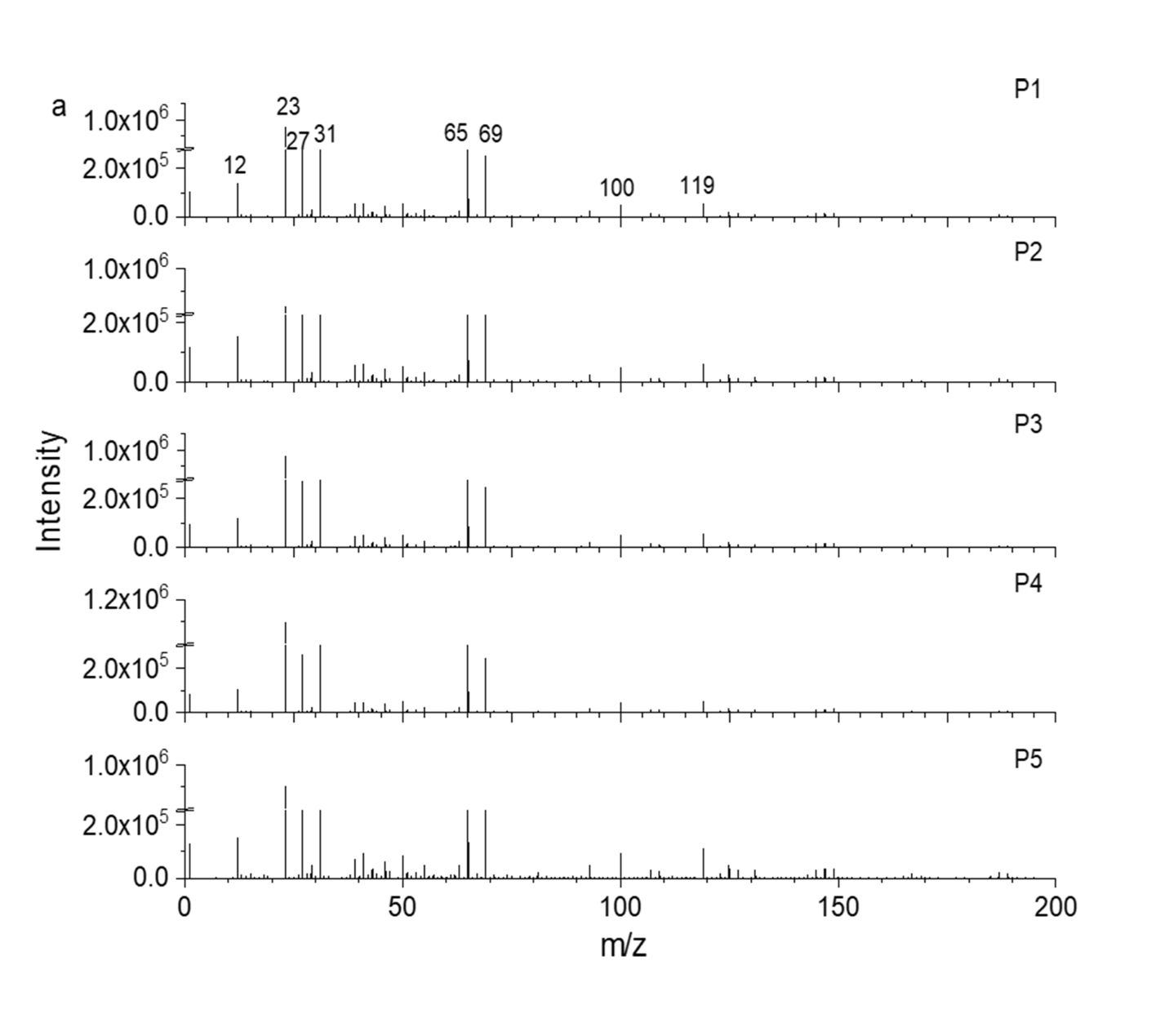


## Figure S4a. Reproducibility of PFBA SIMS spectral analysis in the range of *m/z*^+^ 0 – 200 in the positive mode.

P1, 2, 3, 4, and 5 represent five consecutive measurements of PFBA in the positive ion mode. ToF-SIMS spectral measurements show good reproducibility. Repeatability results are summarized in **Table 3** in the main text.


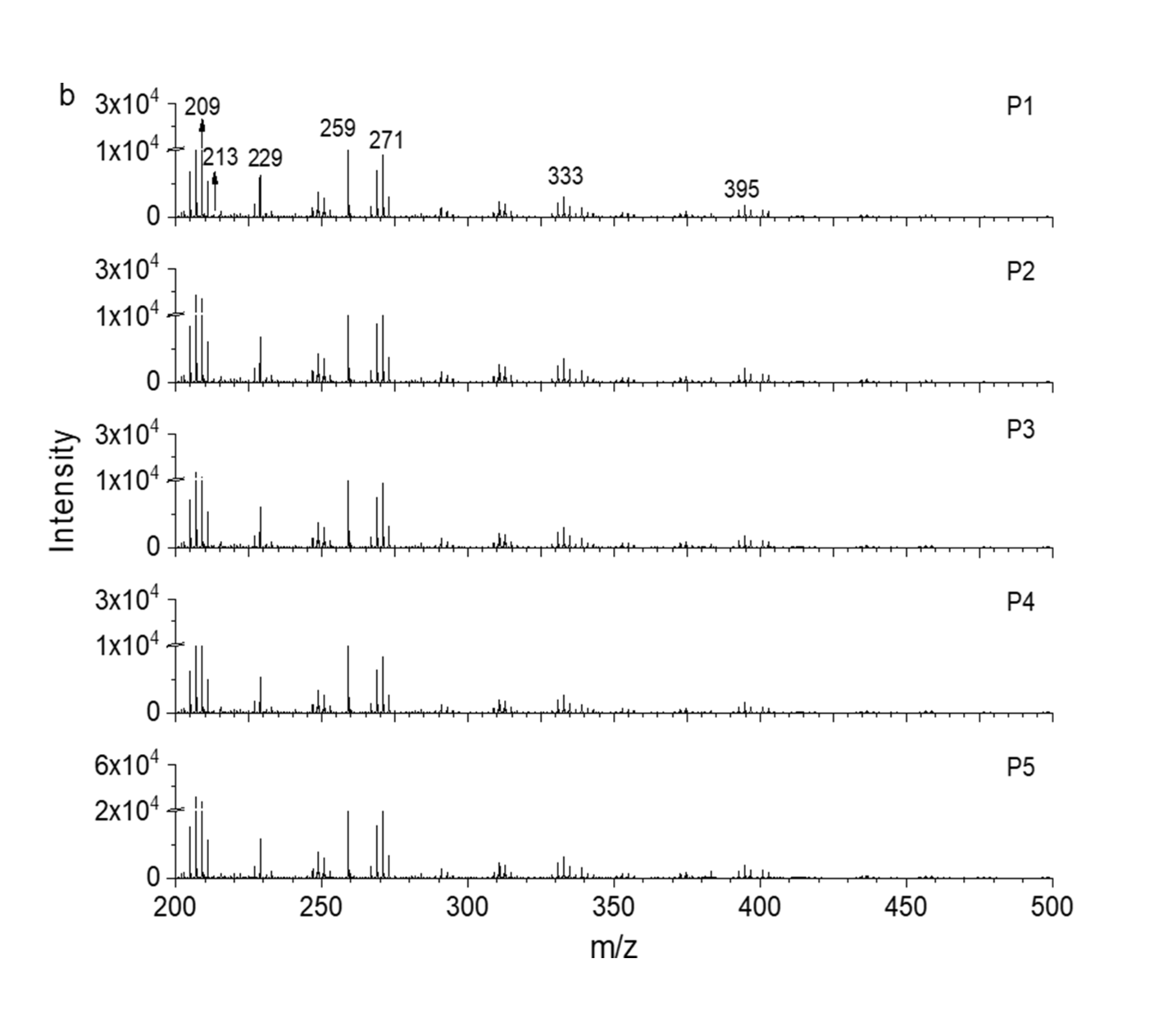


## Figure S4b. Reproducibility of PFBA SIMS spectral analysis in the range of *m/z*^+^ 200 – 500 in the positive mode.

P1, 2, 3, 4, and 5 represent five consecutive measurements of PFBA in the positive mode. ToF-SIMS spectral measurements show good reproducibility. Repeatability results are summarized in **Table 3** in the main text.


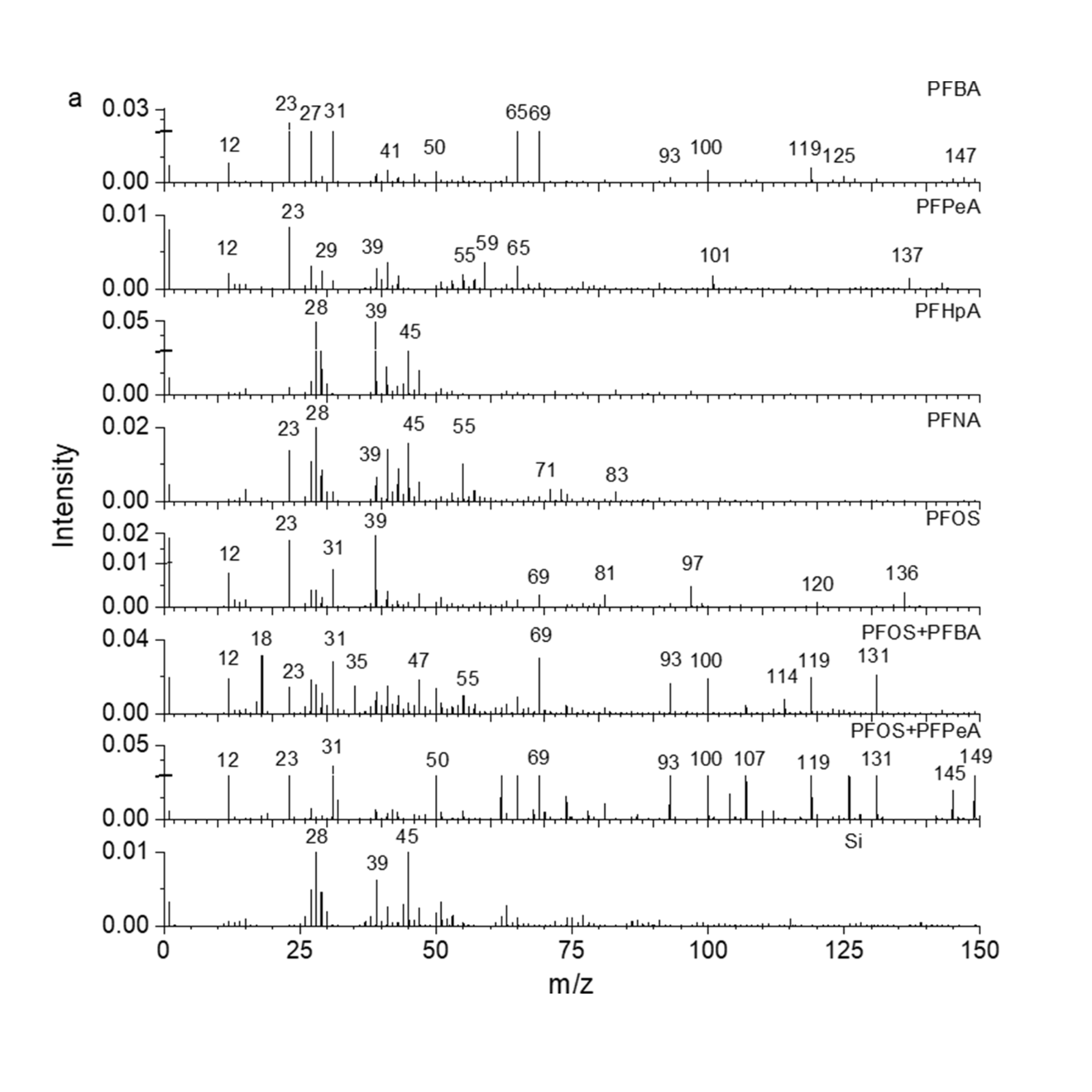


## Figure S5a. SIMS spectral comparison of representative PFASs in the range of *m/z*^+^ 0 – 150 in the positive mode.

PFOS+PFBA refers to the mixture consisting of PFOS and PFBA and PFOS+PFPeA refers to the mixture consisting of PFOS and PFPeA, respectively.


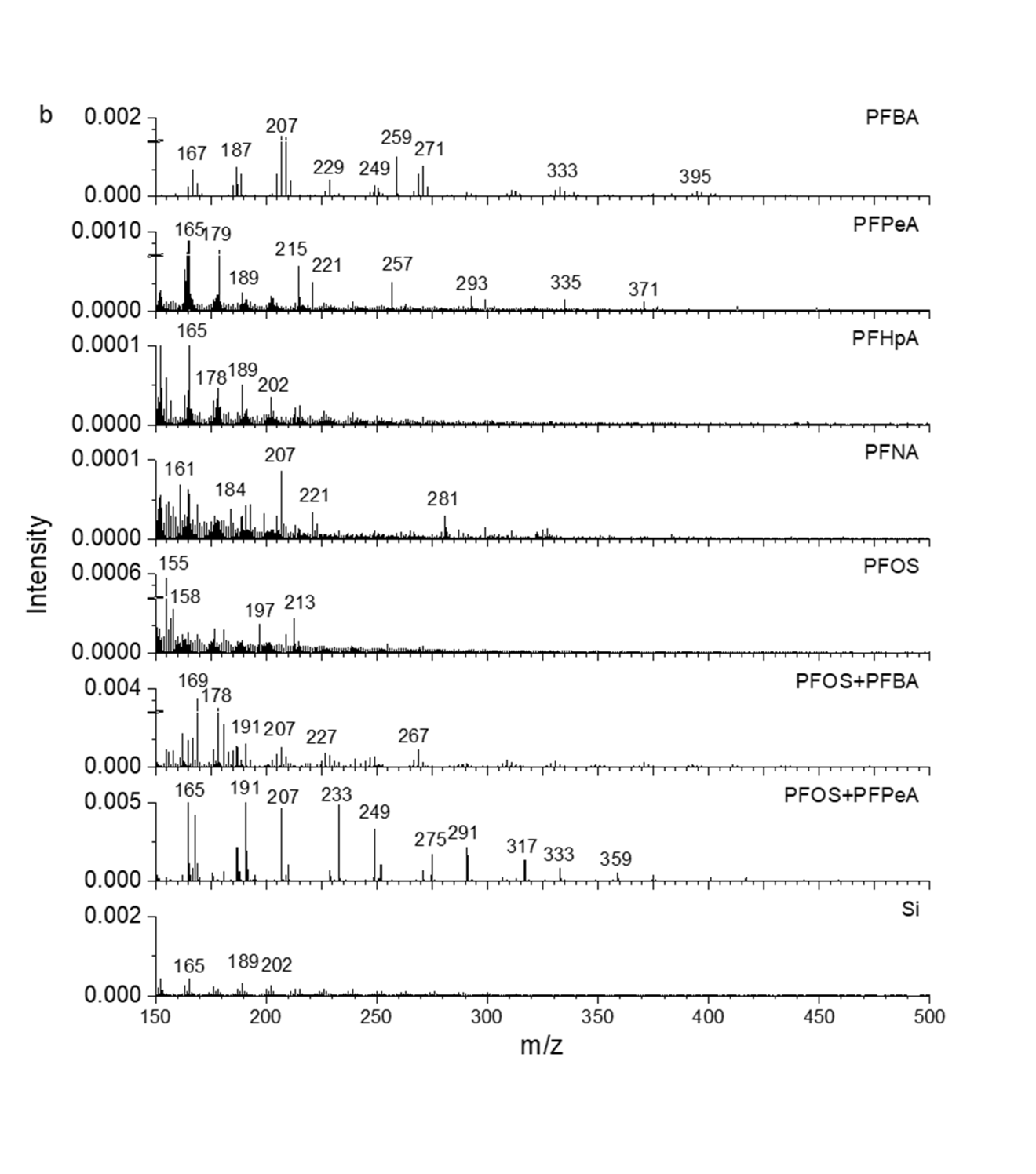


## Figure S5b. SIMS spectral comparison of representative PFASs in the range of *m/z*^+^ 150 – 500 in the positive mode.

PFOS+PFBA refers to the mixture consisting of PFOS and PFBA and PFOS+PFPeA refers to the mixture consisting of PFOS and PFPeA, respectively.

**
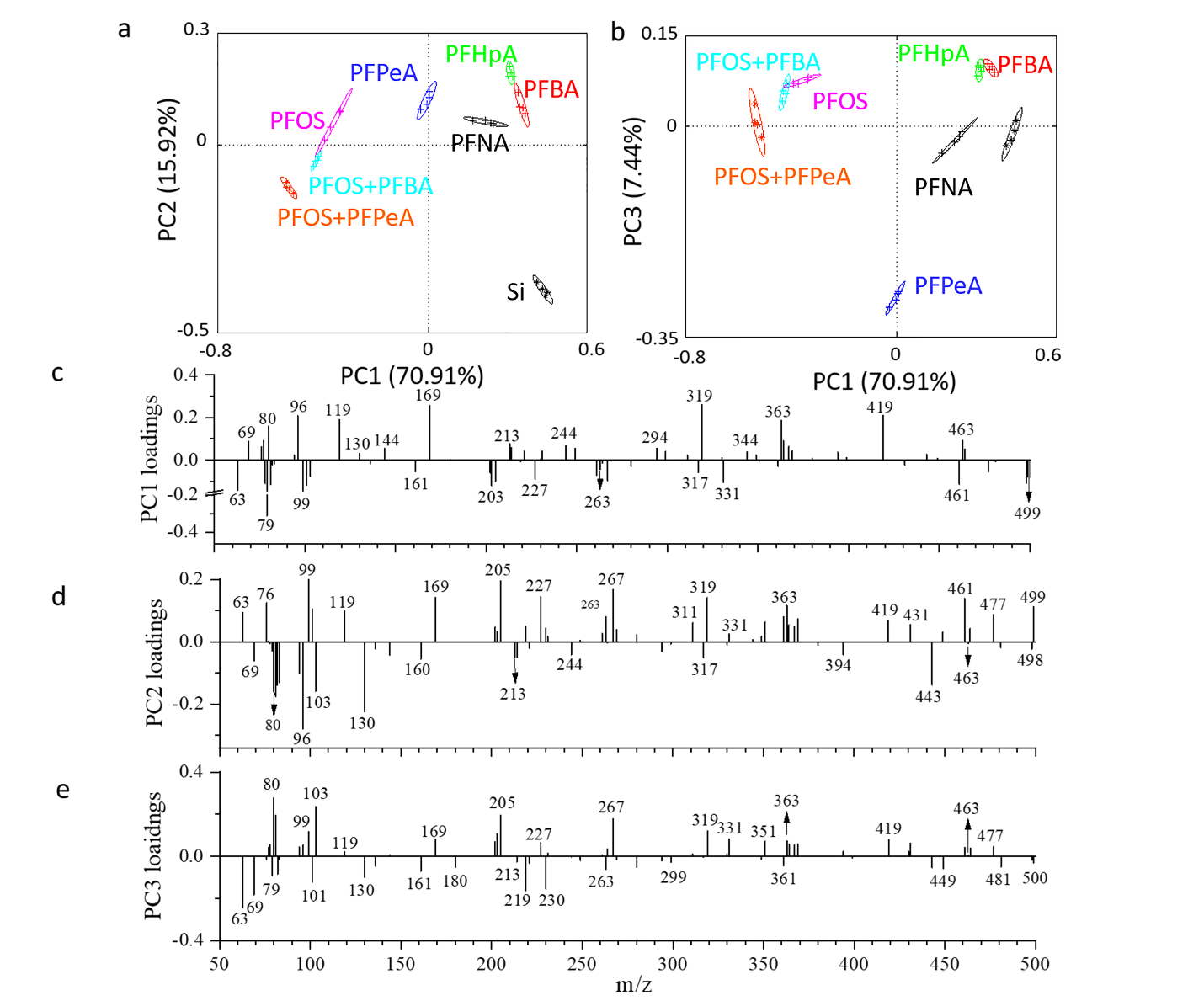
**

## Figure S6. Spectral PCA results of selected peaks in the negative mode: (a) PC1 vs. PC2 scores plot; (b) PC1 vs. PC3 scores plot; (c) PC1; (d) PC2; and (e) PC3 loadings plots.

Representative PFASs analyzed in this work share commonalities in PC1 positive. For example, peaks m/z^−^ 68.995, and 168.994 have high loadings. With molecular weight increase of these PFOA substances, peaks such as *m/z*^−^ 218.9869, 268.980, 318.962, and 418.964 are observed in the reference samples containing PFPeA, PFHpA, and PFNA, respectively. All of them are observed in PC1 positive loadings, consistent with the scores plot in which perfluorocarboxylic acid (PFCA) except PFPeA reside in PC1 positive quadrant.

PC2 separates PFOS and the two mixture samples. It is not surprising that the PFOS molecular peak *m/z*^−^ 498.929 has significant positive loadings in this case. In addition, representative peaks of PFOS such as m/z^−^ 79.969, 98.956, 129.954, and 229.949 as well as the fragment PFCA peaks such as m/z^−^ 68.999, 168.994, 318.962, and 418.964 are observed in PC2 loading plots. It is worth noting that the pseudo-molecular peak *m/z*^−^ 212.968 in the negative PC2 loading comes from PFBA in the mixture sample. The molecular peak *m/z*^−^ 462.970 from PFOS has relatively lower loadings in PC2 negative due the structure commonality between PFNA and PFOS molecules. Similarly, PC3 separates PFPeA from other PFCAs. The molecular peak *m/z*^−^ 263.061 of PFPeA has significant negative PC3 loadings. Molecular peaks of *m/z*^−^ 362.978 and 462.970 of PFHpA and PFNA have positive loadings. The PFOS molecular peak *m/z*^−^ 498.929 can be derived from the mixture of PFOS and PFPeA; while *m/z*^−^ 212.969 with a negative PC3 loading might arise from the PFNA, PFPeA and PFOS mixture due to the molecular structural similarity (Table S1).

The PFNA peak *m/z*^−^ 462.970 has negative PC2 loading and the PFBA peak *m/z*^−^ 212.968 has negative PC2 and PC3 loadings. They are not consistent with the corresponding scores plots. This discrepancy might be attributed to the commonality in the chemical and molecular structures and fragmentation (Table S1). Characteristic ion peaks of PFNA such as *m/z*^−^ 418.964 and 318.962 are situated in the PC2 positive, and typical ion peaks of PFBA such as *m/z*^−^ 168.994 is in PC2 and PC3 positive, which are consistent with their loadings plots, respectively.


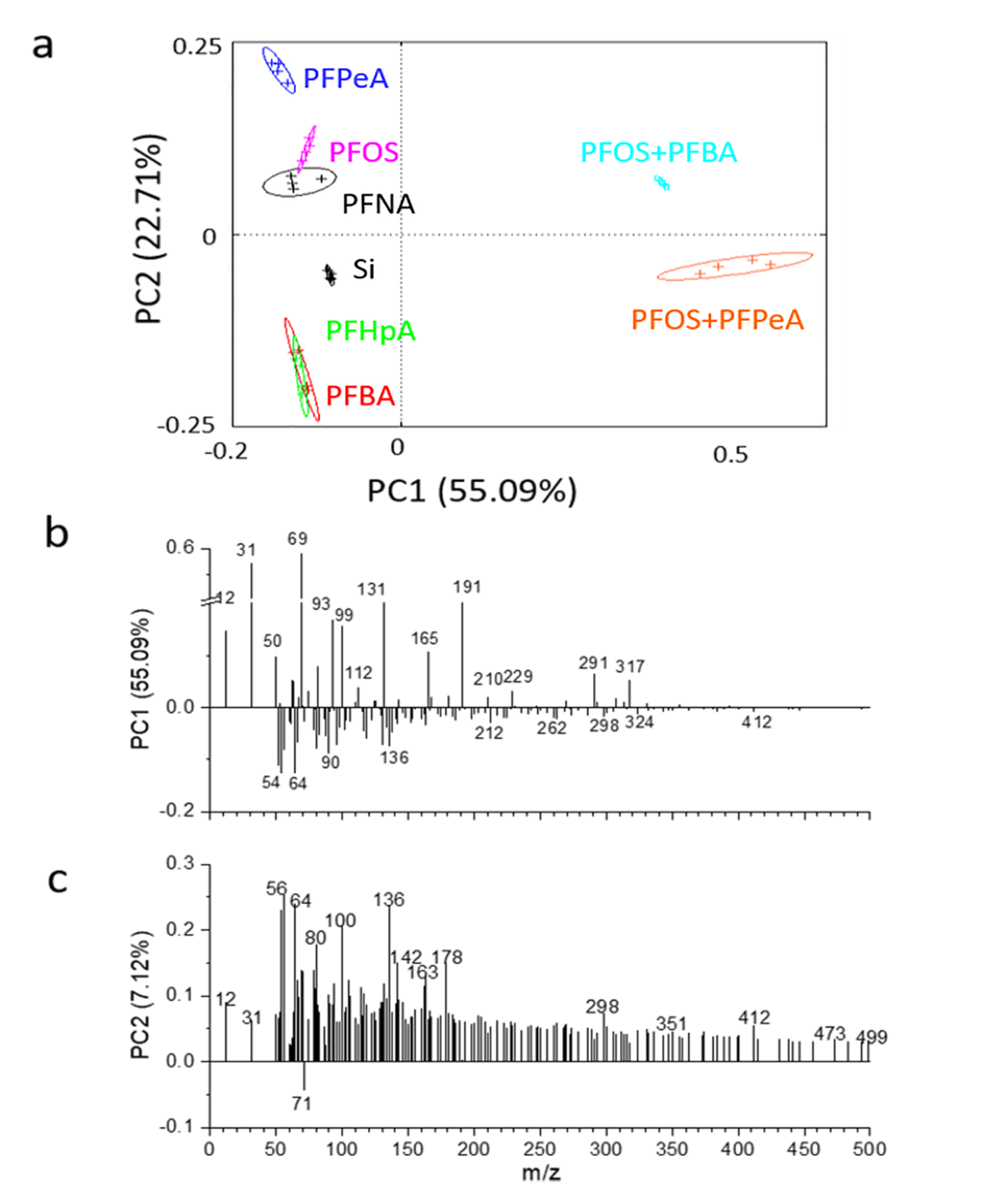


## Figure S7. All peak spectral PCA results of PFOA and PFOS compounds in the positive mode: (a) PC1 vs. PC2 scores plot; (b) PC1 and (c) PC2 loadings plots.

The PFOS+PFBA refers to the mixture consisting of PFOS and PFBA; and PFOS+PFPeA the mixture consisting of PFOS and PFPeA. PC1 separates samples PFBA, PFPeA, PFHpA, PFNA, PFOS and the two mixture samples of PFOS+PFBA and PFOS+PFPeA. PC2 separates two kinds of mixture samples, namely, PFOS+PFBA and PFOS+PFPeA.


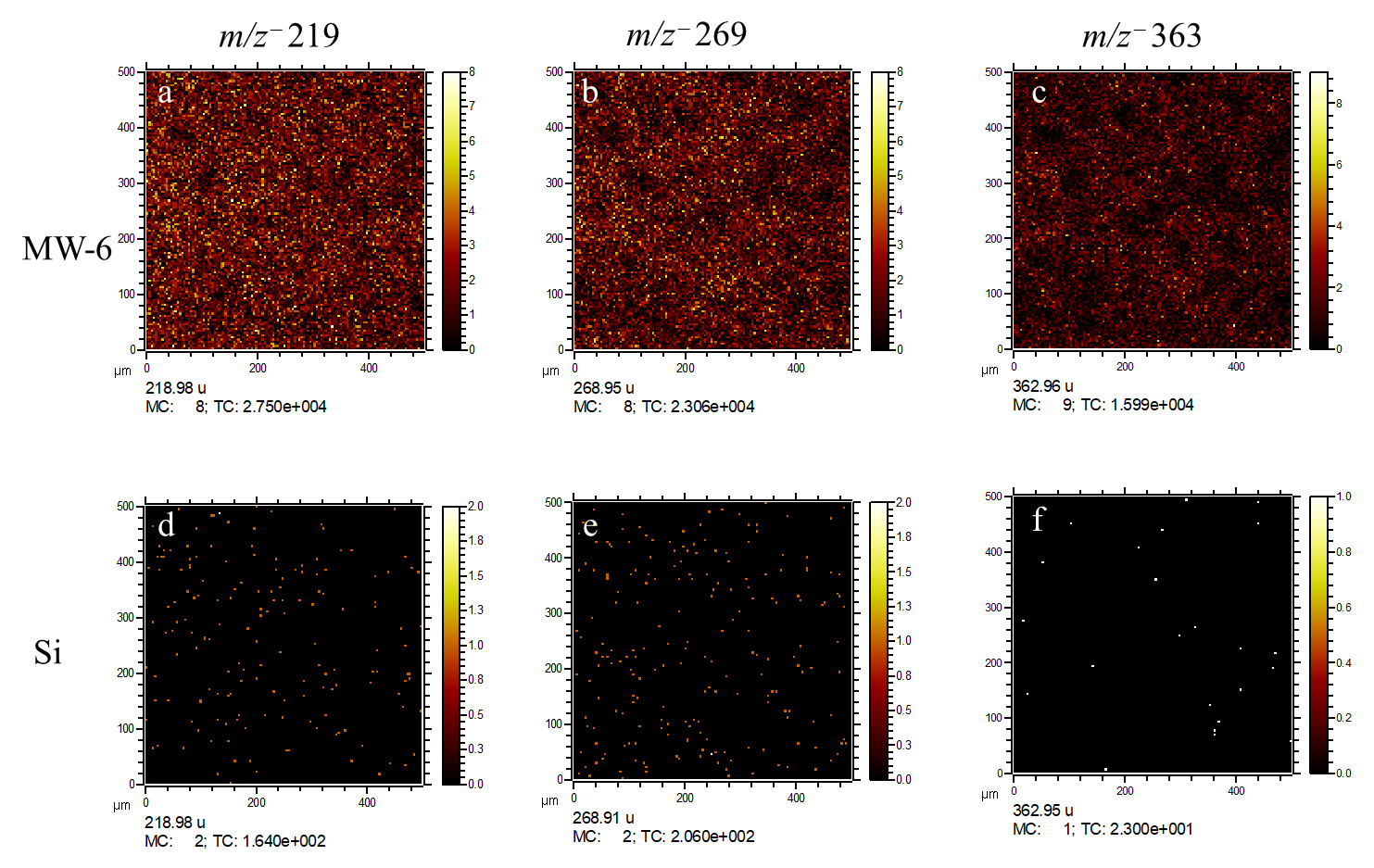


## Figure S8. Comparisons of SIMS 2D images of *m/z^−^* 219, 269, and 363 between the Si wafer substrate (a – c) and the deposited MW-6 ground water sample (d – f), respectively in the negative ion mode.

**Figure S8** depicts the comparisons of SIMS 2D images of *m/z^−^* 219, 269, and 363 between the Si substrate (a – c) and the ground water sample MW-6 (d – f), respectively. Unlike the 2D normalized images in Figs. 3 and 4 in the main text, these results are shown in the measurement counts. The counts of the real-world sample MW-6 are on the order of 10^4^ for peaks of interest. Such intensity indicates that the detected peaks are real and not noise.


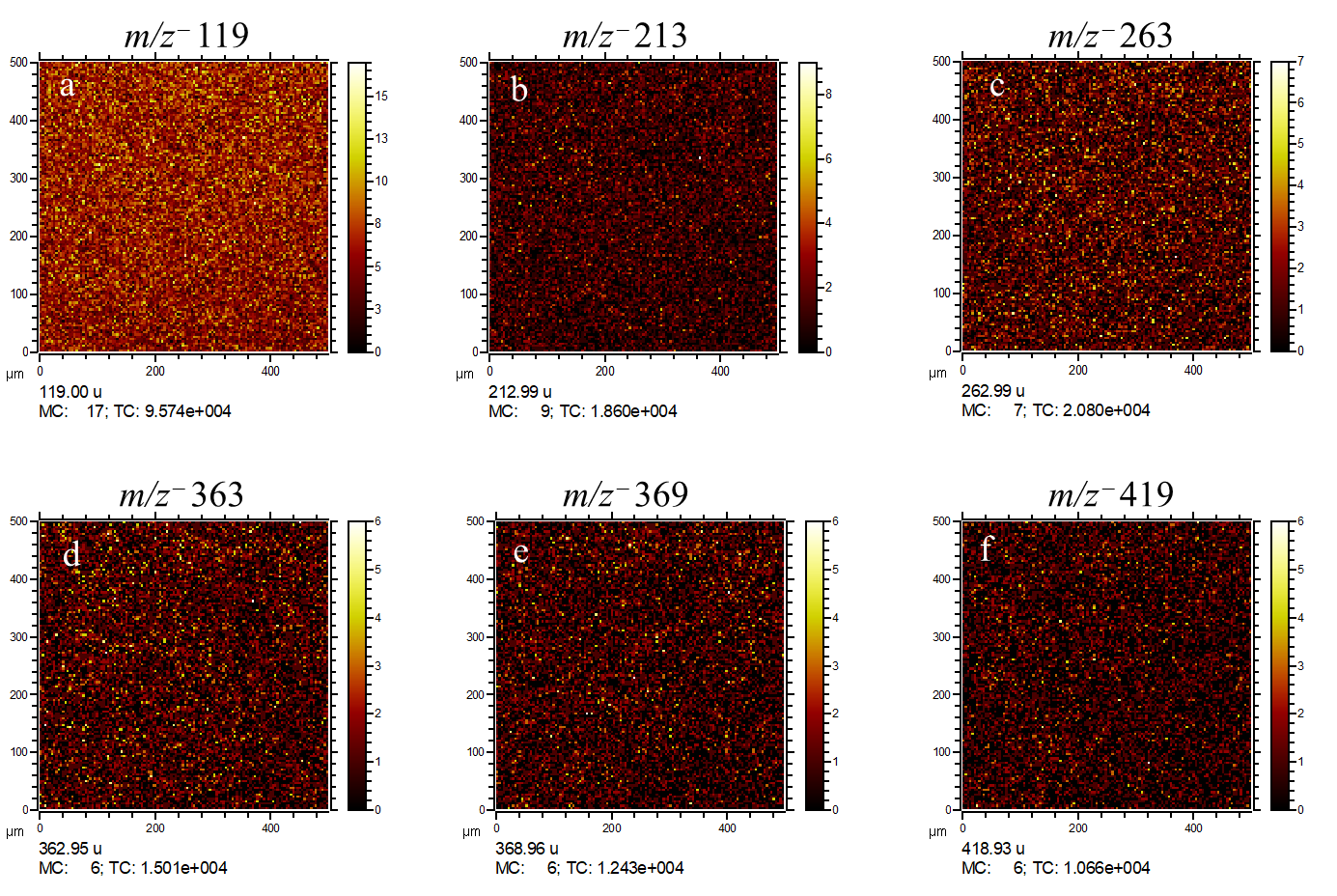


## Figure S9. SIMS 2D images of *m/z^−^* 119 (a), 213 (b), 263 (c), 363 (d), 269 (e), and 419 (f) from the ground water sample MW-5 in the negative ion mode.

SIMS 2D images of *m/z^−^* 119, 213, 263, 363, 269, and 419 of the ground water sample MW-5 (a – f), respectively in **Fig. S9**. Unlike the 2D normalized images in Figs. 3 and 4 in the main text, these results are shown in the measurement counts. The counts of the real-world sample MW-5 are on the order of 10^4^ for peaks of interest. Such intensity indicates that the detected peaks are real and not noise.

# Supplementary Tables

## Table S1. Summary descriptions of representative PFAS reference and mixture samples.

| Sample | Chemical name | Linear formula | MW | Description |
| --- | --- | --- | --- | --- |
| PFBA | Perfluorobutanoic acid | CF_3_(CF_2_)_2_COOH | 213.986 | Liquid form, 0.1 M PFBA |
| PFPeA | Perfluoropentanoic acid | CF_3_ (CF_2_)_3_COOH | 263.983 | Liquid form, 0.1 M PFPeA |
| PFHpA | Pdrfluoheptanoic acid | CF_3_(CF_2_)_5_COOH | 363.977 | Solid form, 0.1 M PFHpA |
| PFNA | Perfluorononanoic acid | CF_3_(CF_2_)_7_COOH | 463.971 | Solid form, 0.1 M PFNA |
| PFOS | Perfluorooctanesulfonate | CF_3_ (CF_2_)_7_ SO_3_H | 499.937 | Liquid form, 0.1 M PFOS |
| PFOS+PFBA | Perfluorooctanesulfonate+ Perfluorobutanoic acid | CF_3_ (CF_2_)_7_ SO_3_H  CF_3_CF_2_CF_2_COOH | NA | Liquid form, 0.07 M PFOS and 0.16 M PFBA |
| PFOS+PFPeA | Perfluorooctanesulfonate+ Perfluoropentanoic acid | CF_3_ (CF_2_)_7_ SO_3_H  CF_3_ (CF_2_)_3_COOH | NA | Liquid form, 0.07 M PFOS and 0.13 M PFPeA |
| Si wafer | Si | Si | 27.977 | Clean Si surface |

**Table S1** shows the sample information including molecular formula, molecular weight (MW), and the main components of each sample analyzed in this work. Each liquid mixture was prepared using deionized water. A drop of 25 µL of the reference material in the liquid mixture was deposited onto a clean silicon (Si) chip for sample preparation. The Si chip containing the reference PFAS materials was dried in the chemical fume hood while protected with Parafilm® overnight prior to ToF-SIMS analysis. When preparing a mixture using reference materials, the two agents, PFOS and PFBA, were mixed with the volume ratio of 1:1, then diluted 50 times with deionized water. The liquid mixture was deposited onto clean Si wafers as described above. The Si wafer was ultrasonically cleaned with acetone, isopropanol, ethanol, and deionized water for five minutes, respectively, and dried in the hood using house nitrogen with a laminar flow.

## Table S2. LODs and LOQs of selected peaks of PFAS compounds.

| *m/z*^−^_obs_ | Suggested Formula | LOD^a^ | LOQ^b^ | Compound |
| --- | --- | --- | --- | --- |
| 168.994 | C_3_F_7_^−^ | 27.97 mg/L | 2.50 mg/L | PFBA |
| 268.980 | C_5_F_11_^−^ | 5.59 mg/L | 2.50 mg/L | PFOS |

^a^LOD: limit of detection. Concentration is based on the SIMS analysis of the PFOS and PFBA solutions, respectively.

^b^LOQ: limit of quantification. Concentration based on the value of the PFOS and PFBA solutions, respectively.

## Table S3. Possible peak assignment of PFOS and PFBA using ToF-SIMS in the positive mode.

| *m/z*^−^_obs_^a^ | *m/z*^−^_the_^b^ | Δm^c^ (ppm) | Suggested Formula | References |
| --- | --- | --- | --- | --- |
| 31.002 | 30.998 | 129.041 | CF^+^ | This work^a^ |
| 49.996 | 49.997 | 20.001 | CF_2_^+-^ | (Langlois et al., 2007)^5^ |
| 68.998 | 68.995 | 43.481 | CF_3_^+^ | (Langlois et al., 2007)^5^ |
| 92.991 | 92.995 | 43.013 | C_3_F_3_^+^ | (Langlois et al., 2007)^5^ |
| 99.989 | 99.994 | 50.003 | C_2_F_4_^+^ | (Langlois et al., 2007)^5^ |
| 111.991 | 111.994 | 26.787 | C_3_F_4_^+^ | This work |
| 130.987 | 130.992 | 38.170 | C_3_F_5_^+^ | (Langlois et al., 2007)^5^ |
| 135.983 | 135.994 | 80.886 | C_5_F_4_^+^ | This work |
| 150.007 | 149.990 | 113.341 | C_3_F_6_^+^ | (Dauchy et al., 2017)^8^ |
| 190.973 | 190.990 | 89.010 | C_8_F_5_^+^ | This work |
| 199.959 | 199.987 | 140.009 | C_4_F_8_^+^ | This work |
| 211.966 | 211.987 | 99.063 | C_5_F_8_^+^ | This work |
| 297.984 | 297.981 | 10.068 | C_9_F_10_^+^ | This work |
| 317.007 | 316.979 | 88.334 | C_9_F_11_^+^ | This work |
| 430.976 | 430.967 | 20.883 | C_9_F_17_^+^ | This work |

^a^*m/z*^−^_obs_: observed mass to charge ratio in the negative ion mode.

^b^*m/z*^−^_the_: theoretical mass to charge ratio in the negative ion mode.

^c^Δm: = Abs (10^6^ × (*m/z*^−^_obs_- *m/z*^−^_the_)/ *m/z*^−^_the_) (expressed in ppm) (Gilmore and Seah, 2000)

## Table S4. Static ToF-SIMS measurement repeatability of PFBA representative peaks in the positive and negative mode.

|  | *m/z*^+^_obs_^a^ | Mean Peak Area^b^ | Area S.D.^c^ | RSD%^d^ |
| --- | --- | --- | --- | --- |
| Positive mode | 30.993 | 5680.7 | 87.0 | 1.53 |
|  | 64.975 | 12221.6 | 157.9 | 1.29 |
|  | 68.988 | 8726.7 | 38.1 | 0.44 |
|  | 99.966 | 2078.0 | 32.6 | 1.57 |
|  | 118.972 | 3427.5 | 87.2 | 2.54 |
|  | 124.930 | 1365.2 | 19.5 | 1.43 |
|  | 258.896 | 1915.6 | 167.2 | 8.73 |
|  | 270.829 | 952.3 | 61.5 | 6.46 |
| Negative mode | 69.003 | 969.1 | 39.8 | 4.11 |
|  | 80.990 | 4911.7 | 280.1 | 5.70 |
|  | 99.980 | 2192.3 | 2.6 | 0.12 |
|  | 169.016 | 8014.0 | 429.9 | 5.36 |
|  | 226.965 | 12769.0 | 716.6 | 5.61 |
|  | 266.933 | 10537.7 | 669.9 | 6.36 |

a: *m/z*_obs_: observed mass to charge ratio

b: average peak area of replicate samples

c: standard deviation (S.D.) shows measurement repeatability

d: RSD% (relative standard deviation%) =area S.D./mean peak area*100

## Table S5. Static ToF-SIMS measurement repeatability of PFOS representative peaks in the positive and negative mode.

|  | *m/z*^+^_obs_^a^ | Mean Peak Area^b^ | Area S.D.^c^ | RSD^d^ |
| --- | --- | --- | --- | --- |
| Positive mode | 31.002 | 1450.7 | 7.7 | 0.1 |
|  | 49.996 | 369.1 | 1.7 | 0.0 |
|  | 68.998 | 1005.9 | 91.2 | 0.9 |
|  | 92.991 | 505.4 | 69.5 | 1.4 |
|  | 99.989 | 107.5 | 21.1 | 2.0 |
|  | 130.987 | 135.9 | 27.2 | 2.0 |
|  | 135.983 | 2751.1 | 339.0 | 1.2 |
| Negative mode | 79.969 | 10641.9 | 407.4 | 3.83 |
|  | 98.956 | 3936.7 | 96.9 | 2.46 |
|  | 118.987 | 365.6 | 2.5 | 0.68 |
|  | 129.954 | 1474.9 | 87.9 | 5.96 |
|  | 229.949 | 558.9 | 15.6 | 2.79 |
|  | 498.914 | 31919.0 | 184.1 | 0.58 |

a: *m/z*_obs_: observed mass to charge ratio

b: average peak area of replicate samples

c: standard deviation (S.D.) shows measurement repeatability

d: RSD% (relative standard deviation%) =area S.D./mean peak area*100
